# Developmental Genes and Malformations in the Hypothalamus

**DOI:** 10.3389/fnana.2020.607111

**Published:** 2020-11-26

**Authors:** Carmen Diaz, Luis Puelles

**Affiliations:** ^1^Department of Medical Sciences, School of Medicine and Institute for Research in Neurological Disabilities, University of Castilla-La Mancha, Albacete, Spain; ^2^Department of Human Anatomy and Psychobiology and IMIB-Arrixaca Institute, University of Murcia, Murcia, Spain

**Keywords:** hypothalamus, genoarchitecture, patterning, secondary organizers, holoprosencephaly, septo-optic dysplasia, *Shh*

## Abstract

The hypothalamus is a heterogeneous rostral forebrain region that regulates physiological processes essential for survival, energy metabolism, and reproduction, mainly mediated by the pituitary gland. In the updated prosomeric model, the hypothalamus represents the rostralmost forebrain, composed of two segmental regions (terminal and peduncular hypothalamus), which extend respectively into the non-evaginated preoptic telencephalon and the evaginated pallio-subpallial telencephalon. Complex genetic cascades of transcription factors and signaling molecules rule their development. Alterations of some of these molecular mechanisms acting during forebrain development are associated with more or less severe hypothalamic and pituitary dysfunctions, which may be associated with brain malformations such as holoprosencephaly or septo-optic dysplasia. Studies on transgenic mice with mutated genes encoding critical transcription factors implicated in hypothalamic-pituitary development are contributing to understanding the high clinical complexity of these pathologies. In this review article, we will analyze first the complex molecular genoarchitecture of the hypothalamus resulting from the activity of previous morphogenetic signaling centers and secondly some malformations related to alterations in genes implicated in the development of the hypothalamus.

## Introduction

The hypothalamus is a highly complex brain territory held to regulate homeostasis and multiple visceral and somatic functions, many of them mediated by the pituitary gland (Saper and Lowell, 2014; Placzek et al., 2020). Because of the remarkable structural heterogeneity of the hypothalamus, the detailed organization of its intrinsic circuitry related to the brain functions it controls remains imperfectly known. Recently a new scenario of hypothalamic studies has emerged due to a marked paradigm shift from the outdated columnar model of Herrick (1910) to the updated prosomeric model of Puelles et al. (2012) and Puelles and Rubenstein (2015). The latter offers a basic regionalization of the mammalian hypothalamus into dorsoventral (longitudinal) and anteroposterior (transversal) developmental units, centered on the notion of natural hypothalamo-telencephalic neuromeric units (i.e., conceiving the telencephalon and eye vesicles as expanded zonal derivatives of the alar hypothalamus).

The prosomeric model is uniquely consistent with the multitude of brain developmental gene expression patterns accrued during the last 40 years, which were meaningless within the columnar model. It can explain many neurogenetic, axonal navigational and patterning data, and applies in other vertebrates (Puelles, 1995; Croizier et al., 2014; Domínguez et al., 2014, 2015; Santos-Durán et al., 2015, 2016, 2018; Nieuwenhuys and Puelles, 2016; Gonzalez et al., 2017; Schredelseker and Driever, 2020). Several recent monographs present structural and functional vertebrate neuroanatomy, including that of the human brain, based on the prosomeric model (Watson et al., 2010; Striedter, 2016; ten Donkelaar, 2018, 2020; Schröder et al., 2020; Striedter and Northcutt, 2020). The advantage of the prosomeric model compared to older models is that it is causally oriented and greatly aids the experimental assessment of molecular and genetic causal mechanisms involved in normal or pathologic neural development. It accordingly promises to aid significantly advances in system physiology and clinical physiopathology in the molecular era, though progress in this direction is still preliminary because physiologists and clinicians are still little aware of the mentioned paradigm shift.

Studies in animal models are essential to evaluate mutations in regulatory genes implicated in hypothalamic development potentially related to rare endocrine disorders associated with congenital malformations such as holoprosencephaly, septo-optic-dysplasia, and congenital obesity. Experimental animal studies, together with data of human patients and their families, are allowing the identification of relevant genes implicated in hypothalamic development, to assess the risk and progression of these rare diseases, and to evaluate possible treatments (e.g., new drugs or gene therapy). Diagnosis and treatment are two of the main problems of patients affected by *rare diseases* whose origin, in a high percentage (estimated up 72%), is due to the unidentified alteration of one or more genes, most of the patients being children (Nguengang Wakap et al., 2020). Genetic and clinical heterogeneity increases the intricacy of rare diseases or disorders.

For instance, holoprosencephaly (cyclopy), a brain malformation with high clinical variability, is not completely deciphered yet, though we know a number of the genes and a variety of mechanisms involved. A 35–50% of cases are due to chromosomal anomalies such as trisomy 13, whereas up to 25% of cases are non-chromosomal and non-syndromic, associated with specific gene mutations (Dubourg et al., 2004, 2018; Petryk et al., 2015). Most of the known altered genes relate to the signaling pathway of *Shh*, and, to a lesser extent, to the *Nodal* and *Fgf* pathways. All of them participate in the development of hypothalamic and other forebrain regions, as well as of craniofacial structures (Arauz et al., 2010; Mercier et al., 2011; reviewed in Roessler et al., 2018). Further studies of these or other molecules involved in hypothalamic development, illuminating the particular consequences of their selective or combined alterations, will help to understand the causes of these diseases with different clinical phenotypes, as well as their aid in early prenatal detection, which would improve genetic counseling.

Before reviewing how the molecular regionalization of the hypothalamus is established, and the consequences of alterations in the function of genes involved in its development, it is necessary to know where the hypothalamus is located, its limits, and relationships with other forebrain structures. Due to the paradigm shift mentioned above, we will see that these are still somewhat controversial topics.

## The Hypothalamus in a Historic Perspective: The Columnar Model vs. the Prosomeric Model

**Figure 1:**
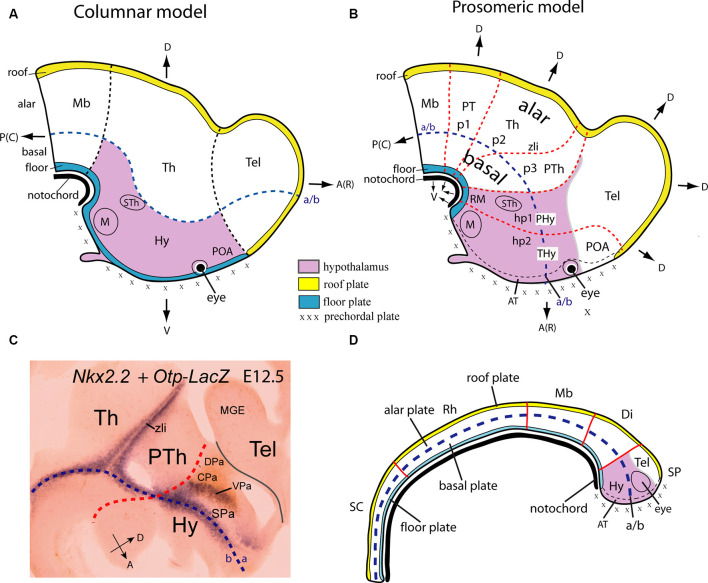
Location of the hypothalamus, and its boundaries with neighboring structures, according to Swanson’s columnar **(A)** and Puelles and Rubenstein’s updated prosomeric **(B)** models. Schemata represent the forebrain at approximately embryonic E16 (human; O’Rahilly and Müller, 1999) and E12.5 (mouse) stages. A Color-code map is indicated. The hypothalamic area is marked in lavender color. In the modified columnar model of Swanson (**A**; 1992, 2003), the hypothalamus, located *caudal* to the telencephalon (Tel) and including the preoptic area (POA) is conceived explicitly as the diencephalic basal plate. By contrast, in the prosomeric model **(B)** the hypothalamus excludes the POA, lies *ventral* to the telencephalon, and *rostral* to the prethalamus (PTh), the rostralmost diencephalic prosomere. The postulated alar-basal boundary (a/b), a typical axial reference, is interpreted differently in these models; it is marked in both **(A,B)** as a blue dash line. Differences between the notions of dorsoventral (D, V) and anteroposterior [or rostrocaudal; A(R), P(C)] spatial dimension are illustrated in both models as well as the color code applied to the extreme longitudinal zones or plates: roof (yellow), alar, basal (both uncolored) and floor (blue). Black dash lines in the columnar model **(A)** indicate the postulated limits of the diencephalon (including the hypothalamus) with the midbrain and telencephalon; note the posterior hypothalamus plus other thalamic regions contact the rostrally expanded midbrain (Mb). Red dash lines in **(B)** illustrate transverse interneuromeric boundaries between the transverse prosomeric units (midbrain, Mb; diencephalon with pretectal, thalamic, and prethalamic prosomeres, p1-p3, and alar PT, Th, PTh domains; hypothalamo-telencephalic prosomeres, hp1, hp2). The intrahypothalamic hp1/hp2 border subdivides the hypothalamus into two rostrocaudal halves, the terminal and peduncular domains (THy, PHy), and the a/b limit subdivides it in alar and basal hypothalamic regions. **(C)** *Nkx2.2* expression in a sagittal section of a mouse embryo at E12.5 (blue signal) carrying an immunoreacted *Otp*-*LacZ* construct (brown reaction). The longitudinal *Nkx2.2* positive band overlaps with the alar-basal boundary (blue dash line) except at the orthogonally labeled zona limitans intrathalamica spike (zli), which identifies the transverse thalamo-prethalamic border. The red dash line indicates the transverse prethalamo- (or diencephalo)-hypothalamic boundary, caudal to the *Otp*-positive paraventricular complex (Pa); the thin black line defines the longitudinal telencephalo-hypothalamic boundary. **(D)** Scheme illustrating the early major dorsoventral and anteroposterior subdivisions in the closed neural tube (red lines) and their relationship with the notochord (in black) at approximately embryonic E12 (human) and E9.5 (mouse) stages. Note the epichordal location of the secondary prosencephalon (SP), including the prospective hypothalamus (in lavender), under the prospective, not yet evaginated telencephalon field (Tel). The forebrain tagma comprises midbrain (Mb), diencephalon (Di), and SP in the updated prosomeric model, rostrally to the rhombencephalon (Rh) and spinal cord (SC) tagmata. Other abbreviations: AT, acroterminal area; CPa, central paraventricular subarea; DPa, dorsal paraventricular subarea; Hy, hypothalamus; M, mamillary region; MGE, medial ganglionic eminence; RM, retromamillary region; SPa, subparaventricular domain; STh, subthalamic nucleus; VPa, ventral paraventricular subarea. **(A,B)** Modified from Puelles and Rubenstein (2015), **(C)** modified from Puelles et al. (2012), and **(D)** modified from Puelles and Martinez (2013).

For more than a hundred years, the hypothalamus was regarded as the ventralmost part of the diencephalon. The latter lay between the rostral telencephalon and the caudal midbrain along a straight axis. This so-called *columnar model* was a result of the attempt by Herrick (1910) to extend the longitudinal functional *columns* of the hindbrain (visceral and somatic motor and sensory domains) into the forebrain on the sole basis of sulcal accidents of the brain ventricular surface (Figure 1A). Herrick mainly documented his columnar conception in numerous studies of adult amphibian brains, but others, notably Kuhlenbeck, subsequently expanded this model to other vertebrate brains, including mammals, and partly to embryos (Kuhlenbeck, 1927, 1973). It has survived with minor changes up to recent times (Swanson, 1992, 2012; Alvarez-Bolado and Swanson, 1996), though it has become progressively obvious to recent researchers investigating embryonic gene expression patterns and functions that a correlation of these with *ventricular sulci* is meaningless and provides no basis for causal explanations. In the modern columnar model of Swanson (1992, 2012), the hypothalamus explicitly corresponds to the diencephalic basal plate (continuous rostrally with the supposedly basal subpallium and caudally with the midbrain tegmentum). Accordingly, a motor character is implicitly ascribed to it, despite containing the sensory eyes and the optic chiasma (this is one of the many inconsistencies of the columnar model, which it cannot account for; Swanson, 1992, 2012; and elsewhere, simply does not mention this feature; the paradigm shift resolves this issue, like many others).

**Figure 2:**
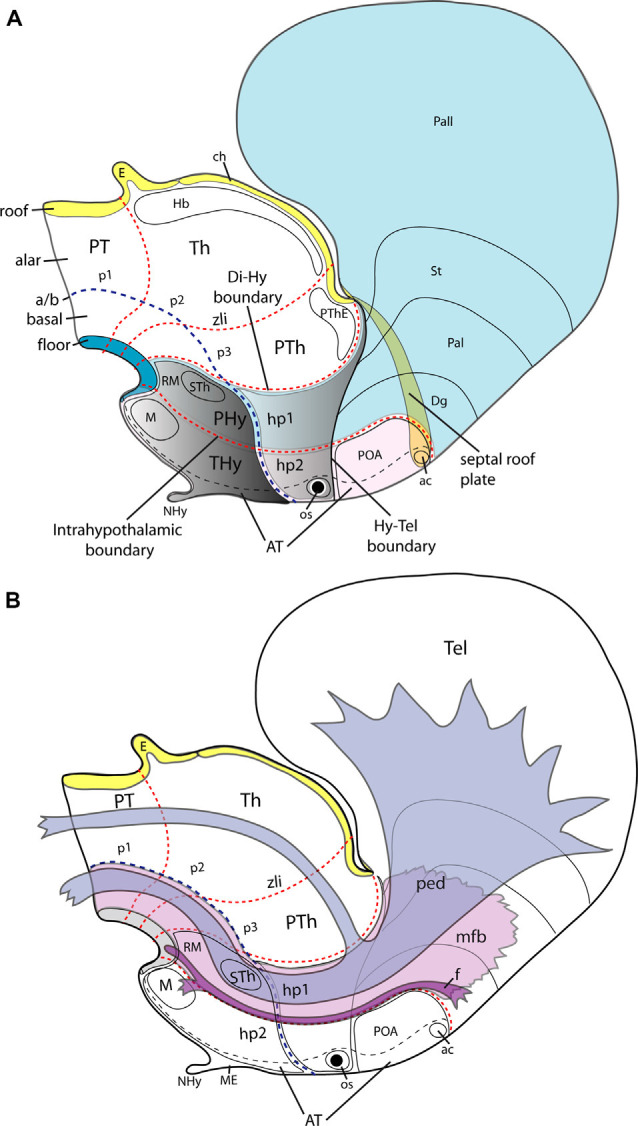
Schemata illustrating the main dorsoventral and anteroposterior subdivisions of the hypothalamus of an E15.5 mouse embryo according to the updated prosomeric model **(A)** and the typical course therein of the peduncular bundles (ped, mfb) and the fornix tract **(**f; **B)**. **(A)** The hypothalamic area is highlighted in gray; it lies rostral to the diencephalic p1-p3 prosomeres, with their respective pretectal, thalamic and prethalamic alar domains (PT, Th, PTh). Transverse red dash lines separate rostrocaudal neuromeric subdivisions. The diencephalo-hypothalamic (Di/Hy) and intrahypothalamic (hp1/hp2) boundaries are particularly indicated. Consequently, the hypothalamus is subdivided into the terminal and peduncular hypothalamic parts (THy, PHy). The acroterminal region (AT), a rostromedial hypothalamic and preoptic formation, including the neurohypophysis (NHy) and optic stalk (os), is delimited by a black dash line (it can be conceived as a singular median hp3 hypothalamo-telencephalic prosomere-see text). The hypothalamo-telencephalic prosomere hp1 contains the PHy plus the evaginated telencephalon (pallial and subpallial subdivisions colored in light blue), whereas the hp2 counterpart contains the THy plus the unevaginated POA (colored in light pink. The largest part of the telencephalon is evaginated and is thus drawn as seen beyond semi-transparent midline structures (septal roof plate and anterior commissure, ac). The roof (yellow), alar, basal (uncolored), and floor (blue) plates are identified. The alar-basal limit is indicated with a blue dash line (a/b) separating the hypothalamus into alar (light grey) and basal (darker grey) parts. Some basal hypothalamic subpopulations are identified as landmarks: mamillary and retromamillary areas /M, RM) and the migrated subthalamic nucleus (STh). The dorsalmost part of the hypothalamus contacts with the telencephalon (Hy-Tel boundary; black dash line). **(B)** The course of tracts associated with prosomere hp1, containing the peduncular hypothalamus (PHy; compare to **A**). The fornix tract (f; in violet) has a dorsoventral course as its sorts out of the telencephalon behind the anterior commissure (ac) and passes successively through the alar and basal peduncular hypothalamus (hp1) to decussate in the retromamillary (RM) floor plate, previously innervating the mamillary body (M) at the basal plate of hp2. The telencephalic peduncle or lateral forebrain bundle (ped, in blue color) and the underlying medial forebrain bundle (mfb; in light violet) have also a transverse dorsoventral course through the PHy; the ped courses next to the caudal hypothalamo-diencephalic border. Once these tracts reach the basal plate they bend backward (around the STh), coursing thereafter longitudinally through the diencephalic, midbrain, and brainstem tegmentum (basal plate). Other abbreviations in **(A,B)**: ch, chorioidal roof; Dg, diagonal subpallial domain; E, epiphysis; Hb, habenular complex; os, optic stalk; Pal, pallidal subpallial domain; Pall, pallium; POA, preoptic area; PThE, prethalamic eminence; St, striatal subpallial domain. Modified from Puelles et al. (2012).

The columnar authors *assume* implicitly (without discussion or any supporting data) that the longitudinal axis of the brain enters the telencephalon (Figure 1A; dash blue line). This contrasts with the curved longitudinal axis of His (1893), his *sulcus limitans*, and subsequent proponents of the prosomeric model, whose forebrain axis is parallel to the cephalic flexure and ends behind the optic chiasma (Figure 1B; Puelles et al., 2012; Puelles and Rubenstein, 2015; Nieuwenhuys and Puelles, 2016; see their Figure 4). The columnar and prosomeric axes are thus orthogonal to each other (part of the paradigm shift). Consequently, we now interpret meaningfully the four traditional diencephalic “longitudinal” *columns* as caudo-rostrally disposed of *transverse* diencephalic and hypothalamic segments (pretectum, thalamus, prethalamus, plus a bipartite -terminal and peduncular- hypothalamus (PT, Th, PTh, PHy, THy; Figures 1B, 2A). These units uniformly display their respective alar and basal domains (solving the problem of the “basal” columnar eyes and chiasma, explained as alar elements). They extend from the diencephalic and hypothalamic floor plate to the corresponding roof plate, both of them being true longitudinal landmarks, like the alar-basal boundary (rather than the columnar ventricular sulci; Puelles, 1995; Nieuwenhuys and Puelles, 2016). Note the hypothalamic roof corresponds to the “telencephalic” septocommissural and chorioidal roof since the hemisphere is a hypothalamic caudal alar evagination (the eye vesicles are smaller evaginations restricted to the rostralmost alar hypothalamus). The hypothalamus thus lies as a whole under the telencephalon and rostrally to the reduced diencephalon within the updated prosomeric model (Figure 1B). The crucial difference between the columnar and prosomeric models is the different conception of the longitudinal axis. In one case, it was defined arbitrarily and teleologically (aiming to explain the forebrain *functionally* as an expanded hindbrain), postulating unwittingly a bifid telencephalic axial end (Herrick, 1910; Swanson, 2012). In the other case, it was based on a modern understanding of fundamental patterning mechanisms linked to early notochordal signals, obtaining an orthogonal hypothalamic end (His, 1893, 1904; Puelles et al., 2012; Puelles and Rubenstein, 2015). This *primary* axial differential feature necessarily modifies the important *secondary* notions of the *dorsoventral* (DV) and *anteroposterior* (AP) dimensions of the brain (compare Figures 1A,B). Using one or the other set of spatial references (columnar vs. prosomeric) has important differential outcomes when interpreting the effects of the multiple signaling molecules on forebrain regionalization in normal and altered development (see below).

The prosomeric forebrain alar-basal longitudinal boundary roughly overlaps the longitudinal lineal expression of *Nkx2.2* along the entire forebrain, now including the midbrain (Figure 1C; Shimamura et al., 1995; Rubenstein et al., 1998; Hauptmann et al., 2002; Domínguez et al., 2011; Puelles et al., 2012, 2020; Nieuwenhuys and Puelles, 2016). The *Nkx2.2*-positive band extends along the alar-basal boundary (a sign of equilibrium between ongoing floor-caused basal ventralization vs. roof-caused alar dorsalization, a fundamental patterning antagonism) before it can be detected by any other means. It also divides the hypothalamus into alar and basal moieties. If we leave aside the telencephalon, which is entirely alar in this model (another part of the paradigm shift) and derives from the “dorsal” alar hypothalamus, the non-telencephalic hypothalamus comprises the remaining “ventral” alar, basal and floor plate longitudinal (dorsoventral) domains (Figures 1B, 2A; details below).

**Figure 3:**
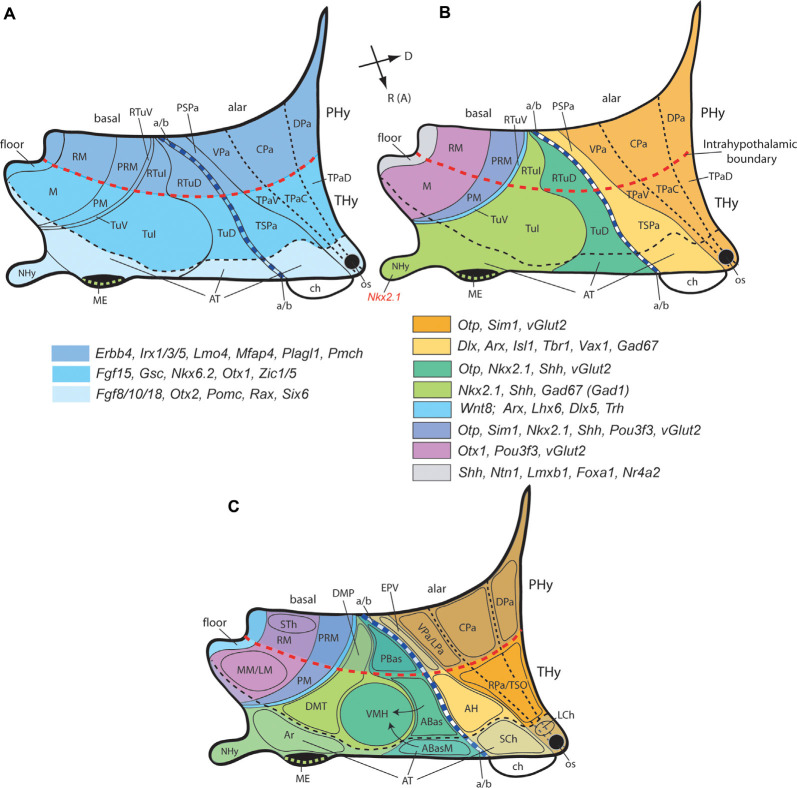
Second level dorsoventral (alar and basal) histogenetic subdivisions of the hypothalamus **(A)**, genoarchitectonic maps **(B)**, and location of main nuclear derivatives **(C)** based on the updated prosomeric model **(**Figure 2**)**. **(A)** The hypothalamus divides rostrocaudally into the terminal and peduncular parts (THy, PHy; dark blue), delimited by the intrahypothalamic boundary (transverse red dash line); the rostro median region of THy constitutes the acroterminal domain (AT; light blue), delimited by a black dash line. The longitudinal alar/basal limit (a/b; thick blue dash line) separates the alar and basal hypothalamus. The alar hypothalamus is subdivided dorsoventrally into paraventricular (Pa) and subparaventricular domains (SPa), each of them having peduncular and terminal components (e.g., PSPa, TSPa), plus the corresponding acroterminal areas. The peduncular/terminal Pa has three DV subdivisions: dorsal, central, and ventral. The basal hypothalamus is primarily subdivided dorsoventrally into tuberal/retrotuberal (Tu/RTu), perimamillary/retroperimamillary (PM/PRM), and mamillary/retromamillary areas (M/RM), plus the corresponding acroterminal parts. The large Tu/RTu area subdivides further into dorsal, intermediate, and ventral parts (TuD/RTuD, TuI/RTuI, TuV/RTuV). Rostral (or anterior; R [A]) and dorsal (D) spatial directions are indicated in **(B)**. **(B)** A schematic color-coded map of characteristic genoarchitectonic patterns is differentially expressed in the hypothalamic dorsoventral subdivisions, based on Puelles et al. (2012) and Díaz et al. (2015). Labels as in **(A)**. **(C)** Map of representative hypothalamic nuclei derived from the molecularly-defined progenitor PHy, THy, and AT domains illustrated in the diagrams shown in **(A,B)**. Arrows represent ventral migrations from the dorsal anterobasal complex from the TuD area (ABas, ABasM), which generation the hypothalamic ventromedial nucleus (VMH) in the TuI area. Other abbreviations: ABas, anterobasal nucleus; ABasM, median anterobasal nucleus; AH, anterior hypothalamic area; Ar, arcuate nucleus, ch, chiasma; DMP, peduncular part of the dorsomedial nucleus; DMT, terminal part of the dorsomedial nucleus; EPV, ventral entopeduncular nucleus; LM, lateral mamillary nucleus; LCh, lateral chiasmatic nucleus; LPa, lateral paraventricular nucleus; ME, median eminence; MM, medial mamillary nucleus; NHy, neurohypophysis; os, optic stalk; PBas, posterobasal nucleus; RPa, rostral paraventricular nucleus; SCh, suprachiasmatic nucleus; STh, subthalamic nucleus; TSO, terminal supraoptic nucleus.

The rostralmost hypothalamo-telencephalic locus, named recently the *acroterminal domain* (AT), occupies the rostral midline (Figures 1B,D, 2, 3; AT; Puelles et al., 2012). Classic studies did not distinguish conceptually this domain. It extends, as shown by experimental fate mapping, from the mamillary rostral end of the floor plate to the prospective anterior commissure site, that is, the rostral end of the septocommissural roof plate (Cobos et al., 2001). Like the rest of the hypothalamus, the AT divides into basal and alar subdomains, separated by the rostral midline confluence of the bilateral alar-basal boundaries. Various unique structures develop at this hypothalamic locus, not present elsewhere in the hypothalamus. For instance, the median eminence and the infundibulum/neurohypophysis complex at the basal AT part, and the optic chiasma and preoptic terminal lamina at the alar AT part; the bilateral retinal cups and optic stalks also are alar acroterminal singularities (Figures 1B,C; Puelles et al., 2012; Puelles and Rubenstein, 2015).

The AT clearly relates causally to sequential “prechordal plate” signals (Figure 1), whose nodal mesodermal cellular sources migrate actively from the rostralmost floor neighborhood (in front of the notochord) up to a neighborhood above the rostralmost roof. Note that in the updated prosomeric model the prechordal plate accordingly is not a *fixed* “plate,” nor represents an axial dimension (i.e., it does not extend *rostrally* to the notochord, as was classically assumed in the columnar model). Prechordal migrating cells do not induce at the median AT the equivalent of a notochordal floor plate. Instead, the prechordal cell population moves progressively along the prospective AT in a dorsalward progression from the floor to the roof of the neural primordium. The prechordal plate population induces heterochronic specific effects along this course in the basal and alar hypothalamic/telencephalic AT and surrounding less rostral areas. For instance, prechordal signals specify the mamillary body in the basal hypothalamus (García-Calero et al., 2008), but separate the right and left eyes from the primarily median eye field across alar AT (by repressing the eye fate at the midline). Malfunction of this last mechanism leads to cyclopic and/or holoprosencephalic syndromes, depending on the spatiotemporal circumstances; obviously, telencephalic effects and even effects on the nasal organs are caused higher up along the alar plate and above the roof plate. In human embryos, a narrow contact occurs between prechordal cells and the chiasmatic field at the hypothalamic alar level at stage 9 (1–3 somites; Müller and O’Rahilly, 2003).

At the neural plate and early neural tube stages, the rostral end of the notochord first contacts intimately and induces the hypothalamic floor plate, corresponding to the prospective retromamillary and mamillary floor regions. There is no further floor rostral to the mamillary floor because the notochord does not extend in that direction (the notochord forms in rostrocaudal direction, because it is deposited continuously out of the node as the latter dynamic phenomenon moves back towards the tail). The inducing contact of its tip with the hypothalamic floor is lost subsequently due to the rigidity of the notochord and the formation of the cephalic axial flexure caudally to the hypothalamus, apparently caused by differential alar surface growth vs. restricted basal growth of the neural wall (most of the brain derives from the alar plate).

The notochordal induction in the median overlying neuroectoderm of a floor plate differentiation is a general feature occurring throughout the brain and is visualized by expression of the *Shh* gene in the inducing and induced tissues (e.g., Ericson et al., 1995a,b, 1998). *Shh* codes for the secreted diffusible inducing protein SHH. Within the ventral forebrain (midbrain, diencephalon, and hypothalamus) diffused notochordal SHH exerts early on its so-called “ventralizing” effect on median ventral cells, specifying them as the prospective floor. This occurs as well in the hindbrain and spinal cord. As a result, the floor plate neuroepithelial cells activate the *Shh* gene and start secreting themselves the SHH morphogen. This diffuses into the neighboring basal plate zone (possibly even into alar domains). Only within the basal plate of the expanded forebrain (down to the isthmus) is the diffused floor SHH signal strong enough to induce locally *Shh* expression (expression remains restricted to the floor plate at hindbrain and spinal cord levels). This characterizes differentially the midbrain, diencephalic and hypothalamic basal plates, and converts them into additional sources of “ventralizing” SHH (Figures 1B,D; Puelles and Rubenstein, 2015; note that, later, part of the tuberal basal hypothalamic Shh expression results in repressed by signals coming from the apposed adenohypophysis).

The epichordal location of the entire forebrain (and associated *rostral* topological position of the mamillary bodies) is accordingly an important novelty emerging in the updated prosomeric model. This has relevant novel implications for interpreting the regionalizing effects of signaling molecules diffusing from various hypothalamic organizing centers, as will be seen below (Puelles et al., 2012; Puelles, 2013, 2017; Puelles and Rubenstein, 2015). Moreover, the prechordal plate, an external *rostral* signaling center* moving* dorsalward along the acroterminal region at early embryonic stages, is not located “ventrally” to the prospective hypothalamus and telencephalon as is usually assumed in columnar literature.

## The Hypothalamus in the Updated Prosomeric Model

The prosomeric model reveals a shared *genoarchitectonic* hypothalamic structure in amniotes and anamniotes (Shimogori et al., 2010; Diez-Roux et al., 2011; Moreno et al., 2012; Puelles et al., 2012, 2013; Puelles, 2013; Domínguez et al., 2014; Morales-Delgado et al., 2014; Díaz et al., 2015; Ferran et al., 2015; Puelles and Rubenstein, 2015; Santos-Durán et al., 2015, 2016, 2018; Schredelseker and Driever, 2020). Affaticati et al. (2015) and Yamamoto et al. (2017) largely confirmed this shared molecular pattern in zebrafish but suggested an alternative vision of the preoptic domain. There is also a genoarchitectonically similar hypothalamus in prechordates (Albuixech-Crespo et al., 2017a,b). The rich genoarchitectonic pattern observed finds no explanation or useful application within the outdated columnar model.

Such comparative studies have generated a first molecular map of distinct hypothalamic alar and basal progenitor domains, quickly expanding it with novel data (e.g., Morales-Delgado et al., 2011, 2014; Puelles et al., 2012; Díaz et al., 2015; Ferran et al., 2015). Different progenitor domains display singular combinatorial profiles with dozens of active and repressed transcription factor genes, partly shared (Puelles and Ferran, 2012). These sets of genomically active transcription factors select among distinct regulatory pathways in the genomic network and enable the local matrix cells to regulate differential aspects of proliferation, produce specific classes of fixed or migrating neuronal types, and modulate other local histogenetic peculiarities affecting axonal navigation and synaptogenesis (Nieuwenhuys and Puelles, 2016). Using this progressively enriched molecular map, we can now start to explore the causal mechanisms leading to comparable neuronal derivatives across vertebrates.

The hypothalamus, which includes the neurohypophysis in its basal acroterminal subdomain, is located ventral to the telencephalon and rostral to the diencephalon proper (Figures 1, 2; see Puelles et al., 2012; Puelles and Rubenstein, 2015 for details). It develops from the secondary prosencephalon, the rostralmost region of the brain, which produces jointly hypothalamus, eye vesicles, and telencephalic hemispheres. Recent genoarchitectonic and fate maps do not ascribe the preoptic area (POA) to the hypothalamus, but to the telencephalic subpallium, as was thought initially (His, 1893, 1904; Bulfone et al., 1993; Rubenstein et al., 1998; Puelles et al., 2000, 2004, 2012; Flames et al., 2007; Bardet et al., 2010; Shimogori et al., 2010; Gelman et al., 2011; Medina and Abellán, 2012).

As regards hypothalamic molecular limits with neighboring regions (telencephalon and diencephalon), the *Dlx*, *Arx*, and *Mash1* gene markers of the telencephalic subpallium (basal ganglia) stop ventrally at the dorsal limit of the alar hypothalamus, where differential markers such as *Otp* and *Sim1* are characteristic. This defines the longitudinal dorsoventral (DV) boundary of the hypothalamus with the telencephalon (Hy-Tel boundary; Figure 2A; Fan et al., 1996; Puelles et al., 2012; note this is more conventional than real since the telencephalon is an evaginated alar hypothalamic derivative emerged in agnathans; a Sim1-expressing part of the alar hypothalamus co-evaginates into the telencephalic vesicle). The transverse hypothalamo-diencephalic boundary lies caudal to the peduncular hypothalamus, coinciding with the caudal end of *Otp* and *Sim1* expression at the alar hypothalamic *paraventricular area* and the basal *periretromamillary area* (Di-Hy boundary; Figure 2A; Shimogori et al., 2010; Morales-Delgado et al., 2011; Puelles et al., 2004, 2012). Other alar gene markers such as *Rgs4*, *Lmo4*, and *Mfap4* corroborate the same limit (Ferran et al., 2015). Expression of *Plagl1*, *Erbb4*, and *Irx1* in the basal peduncular hypothalamus also stops caudally at the Di-Hy boundary. Moreover, the expression of members of the *Dlx* gene family in the prethalamus (alar rostral diencephalon) partially stops at the molecular hypothalamo-diencephalic boundary (Puelles et al., 2020).

Note that in the prosomeric model the hypothalamus is separated from the midbrain by the intercalated diencephalon proper (devoid of the hypothalamus), whereas in the columnar model it was assumed without clear-cut evidence that a part of the basal hypothalamus (called “posterior” hypothalamus) extended caudalwards “under” the thalamus to contact the midbrain tegmentum. We now interpret within the prosomeric model this classic columnar bridge region as the diencephalic basal/floor plates or tegmentum.

During early development, the hypothalamic primordium in vertebrates subdivides molecularly along the prosomeric anteroposterior and dorsoventral dimensions, created by the intersection of the new limits several progenitor domains, each characterized by a differential gene expression code (Shimogori et al., 2010; Diez-Roux et al., 2011; Puelles et al., 2012; Domínguez et al., 2015; Ferran et al., 2015; Santos-Durán et al., 2015, 2016, 2018; Schredelseker and Driever, 2020). As mentioned above, the hypothalamus subdivides *dorsoventrally* into alar, basal, and floor longitudinal fields throughout its length; the dorsalmost alar subregion corresponds to the telencephalic evagination (which is thus by origin entirely alar, irrespective of its subsequent pallio-subpallial subdivision). Also, the hypothalamus subdivides *caudorostrally* into two transverse neuromeric areas, the caudal *peduncular hypothalamus* (PHy) and the rostral *terminal hypothalamus* (THy). Both are continuous dorsally with specific parts of the hemisphere and the telencephalic roof plate (neuromeres are by definition “complete” transverse divisions of the neural tube, extending from floor to roof. PHy and THy represent thus the hypothalamic parts of the complete hypothalamo-telencephalic prosomeres htp1 and htp2 (normally abbreviated hp1, hp2). The whole evaginated telencephalon corresponds to PHy and hp1, whereas the non-evaginated telencephalic POA corresponds to THy and hp2 (Figure 2).

The novel terms “peduncular” and “terminal” hypothalamus (introduced by Puelles et al., 2012) intend to reduce the confusion created by the columnar-to-prosomeric shift in the conceptual paradigm, by referring to features clearly observable in all vertebrates. The massive lateral and medial forebrain bundles of the cerebral peduncle, together with the fornix tract, ostensibly course dorsoventrally through PHy (or hp1) before the cerebral peduncle bends caudalwards into the tegmentum, hence the chosen “peduncular” name for this caudal part of the hypothalamus (Figure 2B; Puelles et al., 2012). The descriptor “terminal” refers to the characteristic topological position of THy at the rostral “terminus” of the neural tube. In the mouse, PHy and THy are characterized by the differential expression of selective molecular markers such as, e.g., *Fgf15*, *Gsc*, *Nkx6.2*, *Otx1*, and *Zic1/5* observed within THy and *Erbb4*, *Irx1/3/5*, *Lmo4*, *Mfap4*, *Plagl1*, and *Pmch* found expressed within PHy, although all of them show differential distributions along the dorsoventral axis. These markers collectively define the *intrahypothalamic boundary* (Figures 2A, 3A; Ferran et al., 2015), which coincides with the interneuromeric boundary between hp1 and hp2.

The hypothalamus has no rostral neural neighbors since it represents the most rostral part of the neural tube, jointly with its telencephalic derivatives. As a consequence, the lateral walls of the neural tube singularly fuse one into another at the rostral *acroterminal area* (AT; Puelles et al., 2012; see also Puelles and Rubenstein, 2015). Note the AT also extends into the median part of the POA, where the thin terminal lamina develops. The latter ends dorsally at the anterior commissure, whose bed represents the septo-commissural roof plate domain corresponding to hp2; the rest of the telencephalic median septum and commissures correspond to the roof plate of hp1 (Figure 2A; Puelles et al., 2012). Obeying to the close range relationship of the acroterminal domain with the migrating prechordal cells and their signals, mouse gene markers such as *Fgf8/10/18*, *Otx2*, *Pomc*, *Rax*, and *Six6* are selectively expressed in this rostromedial hypothalamic area, delimiting it from the rest of THy, although with distinct differences along the dorsoventral dimension (Ferran et al., 2015). Though AT was conceived as a special part of hp2 or THy, its distinct molecular profile suggests it might be conceived alternatively as an atypical but still bilaterally symmetric hp3 hypothalamic prosomere; the AT exists already in prechordates (Albuixech-Crespo et al., 2017b).

Placzek and Briscoe (2005), Manning et al. (2006), Fu et al. (2019) and Placzek et al. (2020) misinterpreted in our opinion *Shh*-expressing cells observed in midsagittal sections at the chicken rostral diencephalic ventral midline as an “anterior floor plate” (no doubt assuming wrongly that *Shh* is always a floor plate marker). Actually, these median cells correspond to the *Shh*-expressing *basal* *acroterminal* subdomain. Placzek and collaborators (Fu et al., 2019; Placzek et al., 2020) seem to have assimilated this particular notion, but conjecture a mixture of columnar and prosomeric notions in the form of a novel “anisotropic model of basal hypothalamic development.” Why the model should attend only to basal development remains unclear. In general, these authors seem to imagine the whole hypothalamus as represented by its midline. We now know that part of that midline is an authentic notochord-induced floor plate and the rest is acroterminal domain. What happens at the midline does not explain what happens in the rest of the hypothalamic wall.

As mentioned above, the *forebrain basal plate* generally expresses *Shh* secondary to floor plate production of sufficient SHH signal (Puelles et al., 1996, 2012). The acroterminal basal *Shh-*expressing area is conceivably enlarged by added external prechordal signals; notably, in this respect, the DV dimension of the basal AT area is larger than in any other place in the brain. Placzek and collaborators, possibly misguided by the columnar model (whose expectation of an “anterior floor plate” reaching the telencephalon was not fulfilled in any case), were not consistent in their interpretation with the parallel notion that a floor plate only exists were an early contact of median neuroepithelium with the notochord first occurs. This circumstance is absent at the acroterminal domain, and, accordingly, there is no forebrain floor beyond the mamillary area. This is one of the reasons making the “acroterminal” concept necessary.

Let us return now to PHy and THy dorsoventrally subdivided into longitudinal domains. The alar hypothalamus domain subdivides into parallel *paraventricular* and *subparaventricular* longitudinal areas, which extend through both PHy and THy, as repeatedly observed in several vertebrates (Moreno et al., 2012; Puelles et al., 2012; Domínguez et al., 2013; Ferran et al., 2015; Santos-Durán et al., 2016). The paraventricular area (Pa) is the dorsalmost hypothalamic alar longitudinal domain, being significantly broader dorsoventrally in PHy than in THy (where it contacts with the telencephalic POA). The Pa area is an *Otp*/*Sim1*-positive and *Dlx*/*Arx*-negative domain whose neurons are mostly glutamatergic and produce a series of peptidergic products (Puelles and Rubenstein, 2003; Puelles et al., 2004, 2012; Shimogori et al., 2010; Morales-Delgado et al., 2011; Puelles et al., 2004; Díaz et al., 2015). Neurons produced in this large bi-neuromeric area (hp1 and hp2) are known to project *via* a compact dorsoventral tract along the transverse AT/THy border into the median eminence and the neurohypophysis, establishing the classic supraopto(preopto)-hypophyseal pathway (note some classic authors included the Pa area in the POA, then thought to be hypothalamic). *Rgs4* is restricted to peduncular Pa whereas *Fgf15* characterizes mainly terminal Pa (Ferran et al., 2015).

The subparaventricular area (SPa) lies underneath the Pa and is considerably broader dorsoventrally at the THy than at the PHy. SPa expresses *Dlx*, *Arx*, *Isl1*, *Vax1*, and *Gad67*, and their neurons are mainly GABAergic (Pa; SPa; Figure 3B; e.g., Puelles et al., 2012). Its acroterminal end expresses selectively *Six6* and *Six3* (López-Ríos et al., 2003; Conte et al., 2005), among other markers, and produces a singular paramedian neuronal aggregate, the *suprachiasmatic nucleus* (Puelles and Rubenstein, 2015), know to represent the central circadian clock mechanism (Gillette and Tischkau, 1999; Herzog et al., 2017). The retina and optic stalk area represent evaginated derivatives of the SPa extension into AT, which also appears associated with the optic chiasma (Puelles and Rubenstein, 2015).

In the vertebrate basal hypothalamus, widespread* Shh* and *Nkx2.1* gene markers characterize its ventricular zone and mantle layer, respectively. This pattern occurs across most basal PHy and THy, excepting secondary lack of *Shh* expression at part of the THy tuberal area and absence of *Nkx2.1* at the retromamillary area (Puelles et al., 2012). Other molecular markers are restricted to distinct rostrocaudal prosomeres (PHy, THy, AT) and their dorsoventral basal subdivisions (Figure 3B; Moreno et al., 2012; Puelles et al., 2012; Domínguez et al., 2014, 2015; Ferran et al., 2015; Santos-Durán et al., 2015, 2018; Gonzalez et al., 2017; Schredelseker and Driever, 2020). We distinguish across the bi-neuromeric basal hypothalamus three dorsoventral (longitudinal) domains, a dorsal *tuberal/retrotuberal area* (Tu/RTu), an intermediate *perimamillary/periretromamillary area* (PM/PRM), and a ventral *mamillary/retromamillary* *area* (M/RM; Figure 3). The pairs of entity names refer respectively to correlative THy vs. PHy components (e.g., the tuberal area, Tu, is the terminal basal element that corresponds with the peduncular retrotuberal area, RTu). In general, the terminal Tu and M basal areas are larger than the peduncular RTu and RM ones, while the peduncular PRM area is larger than the terminal PM area (these anteroposterior -AP- differences may be prechordal or AT effects).

The relatively dorsal basal Tu + RTu territory further subdivides dorsoventrally into dorsal, intermediate, and ventral microzonal subdomains, possibly implying analogous DV partitions at the corresponding AT basal domain. This phase of DV regionalization thus defines several tuberal and retrotuberal subareas (TuD, TuI, TuV subareas in basal terminal Tu, and analogous but smaller and less obvious RTuD, RTuI and RTuV subareas in basal peduncular RTu; Figure 3; Puelles et al., 2012; Ferran et al., 2015). We need all of them to place precisely some of the well-known hypothalamic nuclei. This complex picture is complicated further by the existence of numerous tangential cell migrations, both within the basal plate and between the alar and basal plates (Morales-Delgado et al., 2011, 2014; Puelles et al., 2012; Díaz et al., 2015). In some cases, distinct and even massive composite nuclei are found in the adult at sites that are quite different from the microzones where the respective neurons were born (see the cases of the VM and VPM nuclei in Puelles et al., 2012).

Figure 3B summarizes non-exhaustively characteristic gene markers serving so far to identify these primary and secondary basal subdivisions within the prosomeric model (Figure 3B; Puelles et al., 2012; Ferran et al., 2015). The hypothalamic floor plate underlies the retromamillary and mamillary basal plate areas; it is characterized by the expression of marker genes such as *Shh*, *Ntn1*, *Lmxb1*, *Foxa1*, and *Nr4a2* (Figure 3B; Puelles et al., 2012; Ferran et al., 2015; Allen Developing Mouse Brain Atlas).

Each of the described molecularly delimited progenitor subdivisions of the hypothalamus gradually starts its schedule of neurogenesis and usually develops a radial stratification with periventricular, intermediate (sometimes subdivided), and superficial (subpial) mantle cell strata. That is, the microzones or progenitor domains transform into distinct *radial histogenetic areas* (fundamental morphogenetic units of Nieuwenhuys and Puelles, 2016). At this stage novel, gene expression patterns may appear, indicating progressive activation of differentiation genes that control the *differentiation* of the locally derived neuronal types (Puelles et al., 1987, 2004). Intermediate stratum cells particularly of the *peduncular* hypothalamus (both alar and basal) adopt interstitial dispersed positions among the fibers of the medial forebrain bundle and fornix tract, forming what the field conventionally calls *lateral hypothalamus* (Nieuwenhuys et al., 1982; Geeraedts et al., 1990a,b; Puelles et al., 2012; Croizier et al., 2014). It is unclear whether THy participates in the lateral hypothalamus. Early-born cells forming the superficial stratum (directly or *via* tangential migration) constitute adult subthalamic, parasubthalamic, lateral tuberal, tuberal suboptic, supraoptic, and entopeduncular nuclei. Some of these names refer to related tracts, such as the optic tract and/or the lateral forebrain bundle or cerebral peduncle. The subthalamic and parasubthalamic populations tangentially migrate subpially from the RM area into the RTu area (ventrodorsal transposition within the peduncular basal plate). The *sub*- prefix in these two names refer to the outdated columnar axis so that we must translate their descriptive value in the prosomeric model, understanding these hypothalamic elements are actually placed *rostral* to the diencephalic thalamus, rather than *under* it (axial bend at the cephalic flexure). Indeed, the cerebral peduncle covers the subthalamic nucleus just before it turns *caudalwards* into the diencephalic tegmentum (Figure 2B). Inversely, the THy *suboptic* *Otp*-expressing and vasopressin/oxytocin secreting neurons (corrected term introduced by Puelles et al., 2012; they were classically known inconsistently as “tuberal supraoptic neurons”) migrate ventralwards into the superficial Tu region from the alar supraoptic nucleus, whose cells share the same molecular profile. Recently, Alvarez-Bolado and collaborators reported another ventrodorsal subpial tangential migration, which translocates *Foxb1*-expressing cells from the mamillary area into the terminal Pa microzone (Zhao et al., 2008; Alvarez-Bolado and Celio, 2016). The authors described the resulting superficial Parvafox nucleus as part of the “lateral hypothalamus” under the level of the optic tract (suboptic), but it clearly also extends above the optic tract (supraoptic; see *Foxb1* expression at the Allen Developing Mouse Brain Atlas). The lateral tuberal nuclei are characteristic of primates and we know nothing about their developmental origin. The superficial hypothalamus hence forms a complex stratum with distinct (migrated or non-migrated) alar and basal components. In any case, the late-born major hypothalamic cell masses develop within the periventricular stratum of the respective histogenetic areas (the classic so-called “medial” nuclei; Puelles et al., 2012).

Figure 3C illustrates representative hypothalamic nuclei derived from the described histogenetic subregions (see Puelles et al., 2012 for a more detailed stratification). Briefly, the PHy originates major hypothalamic alar structures such as the dorsoventrally subdivided (dorsal, central, ventral) paraventricular nucleus and the ventral entopeduncular nucleus (DPa, CPa, VPa/LPa, EPV), as well as the basal retromamillary area with its migrated subthalamic and parasubthalamic nuclei (RM, STh/PSTh). Non-acroterminal THy instead gives rise to the supraoptic and anterior alar hypothalamic nuclei (TSO, AH), as well as to the enlarged tuberal and mamillary regions that comprise ventromedial, dorsomedial, and medial/lateral mamillary nuclei (VMH, DMP/DMT, MM/LM) as major basal neuronal formations. The acroterminal domain generates its series of specialized alar and basal nuclei or cell populations, some of which migrate dorsoventrally into the acroterminal arcuate nucleus (Morales-Delgado et al., 2011, 2014; Díaz et al., 2015). The optic stalk, the optic chiasm, and the lateral chiasmatic and suprachiasmatic nuclei derive from the AT subparaventricular alar plate whereas the anterobasal, tuberal-suboptic and arcuate nuclei, as well as the median eminence and infundibulum with the neurohypophysis are AT basal derivatives (os, ch, LCh, SCh, TuSbO, Ar, ME, NHy; Puelles et al., 2012).

**Figure 4:**
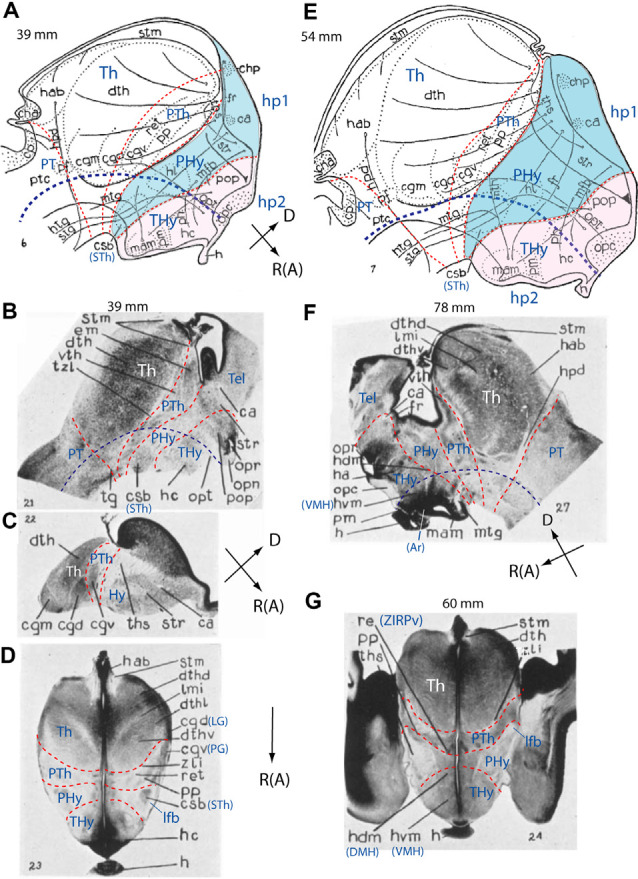
Schemata and microphotographs extracted from the work of Gilbert (1935) on the development of the human hypothalamus at stages of 39 mm **(A–D)**, 54 mm **(E)**, 78 mm **(F)**, and 60 mm **(G)**, with the addition of some prosomeric interpretation details. The reconstruction schemata in **(A,E)** allow distinguishing the diencephalon (PT, Th, PTh) and hypothalamus (PHy; blue background; THy; light pink background; see common spatial orientations at the lower right corner of **A,C,F**). In **(D,G)** rostral (R; or anterior, A) is oriented towards the bottom of the photograph. Tentative interneuromeric boundaries were drawn in as thin transverse red dash lines, and the approximate position of the alar-basal boundary was marked by a thicker dark blue dash line. The original drawings include some fiber tracts and various recognizable anatomic labels. **(A)** Note in our interpretation the *rostral* location of the hypothalamus concerning the diencephalon proper (PT/Th/PTh and corresponding tegmentum). Morphological landmarks such as nuclei and tracts have helped us to demarcate prosomeric subdivisions. Note the fornix (fr) and medial forebrain bundle (mfb) tracts, as well the basal “corpus subthalamicus” (csb; or subthalamic nucleus, STh) are restricted to the PHy. “Mamillaris,” “premamillaris” and “lateral hypothalamic nuclei” (mam, pm, hl), hypophysis (h), and optic chiasma (opc) are located in the THy, which, together with the preoptic region (pop) constitute our hp2 prosomere [the acroterminal (AT) portion was not marked]. **(B,C)** Two brain sagittal sections and a horizontal section extending from the pineal to the chiasma **(D)** through the hypothalamus of 39 mm human embryos. Transverse interprosomeric limits are delineated with red dash lines and pretectal, thalamic, and prethalamic areas of the diencephalon (PT, Th, PTh), as well as peduncular and terminal hypothalamus (PHy, THy), are identified. In **(B,D)** the corpus subthalamicus (csb; or subthalamic nucleus, STh) appears at the basal peduncular hypothalamus (PHy), associated with the lateral forebrain bundle or peduncle (lfb; compare ped in Figure 3B). **(E)** Schema illustrating the main dorsoventral and rostrocaudal prosomeric subdivisions in a 54 mm human embryo. Peduncular and terminal hypothalamus (PHy, THy), included in hypothalamo-telencephalic prosomeres hp1 and hp2, are highlighted in blue and light pink, respectively. See landmark details in **(A)**. Note the larger basal THy (tuberomammillary region) compared to the basal PHy. **(F,G)** Sagittal **(F)** and horizontal (**G**; passing through pineal and hypophysis) sections of two older human embryos (78 mm and 60 mm, respectively). Transverse interprosomeric and longitudinal alar/basal limits were added using the same color-code as in previous figures. The mamillary (mam), arcuate (Ar), dorsomedial (hdm/DMH), and ventromedial (hvm/VMH) nuclear derivatives are identified in the basal terminal hypothalamus (THy), and the anterior hypothalamus and optic recess (opr) in the alar peduncular hypothalamus (PHy). LG, lateral geniculate nucleus (or cgd); PG, pregeniculate nucleus (PG; or cgv); Hy, hypothalamus; Tel, telencephalon; ZIRPv, zona incerta rostral periventricular. See other abbreviations in Gilbert (1935). **(A–G)** Correspond with Gilbert’s Figures 6, 21, 22, 23, 7, 27, and 24.

In humans, morphological and molecular studies on hypothalamic development are scarce. The best classic source is the atlas of the human brain development of Hochstetter (1919, 1923, 1929). Gilbert (1935), a pupil of Papez, published a detailed analysis of the early development of the human diencephalon, including descriptions of the hypothalamus. This study shows coronal and sagittal sections, presenting sagittal view schemata of landmark tracts correlated with neural populations. These schemata allow us to make a rough neuromeric interpretation of her excellent material (Figure 4; see also Papez, 1940, who elaborated specifically on the hypothalamus). Kuhlenbeck and collaborators also studied between the 19-thirties and fifties the mammalian diencephalon, including the human hypothalamus, using a variant version of the columnar model (Kuhlenbeck and Haymaker, 1949; Kuhlenbeck, 1954, 1973; Christ, 1969). Kuhlenbeck supported the modernly refuted notion of Spatz (1925) that the globus pallidus originates in the hypothalamus and singularly interpreted most of the hypothalamus as an alar plate derivative (i.e., held that the basal plate ends at the mamillary bodies, all the rest being alar; this also has been refuted recently). These other works do not surpass Gilbert’s (1935) report in precision.

Altman and Bayer (1986, 1995) illustrated relevant hypothalamic rat embryonic cell birthday data in high-quality histological material, but interpreted it in a personal variant of the columnar model jointly with many preconceived notions, leading to controversial conclusions. Puelles (1996) reviewed critically some results of this approach. The same authors recently produced a developmental atlas of the human brain in several volumes, which is again worth perusing for its excellent histologic quality, but readers might prefer to eschew odd columnar interpretations of the authors (Bayer and Altman, 2007).

**Figure 5:**
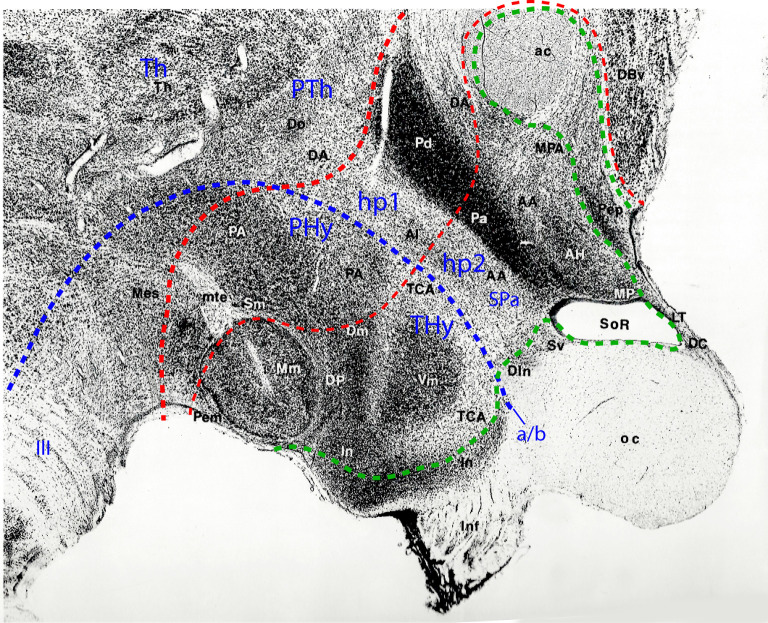
Sagittal paramedian section through the hypothalamus of an adult Rhesus monkey, taken from Bleier’s hypothalamus atlas (Bleier, 1984; her Figure 30), and modified by colored lines parcellating the hypothalamus according to the prosomeric model. Rostral is to the right, dorsal to the upper right. The alar/basal limit indicating the prosomeric forebrain axis is identified with a blue dash line separating the hypothalamus into basal and alar subdivisions. A thin red dash line indicates the interprosomeric limit separating the hypothalamo-telencephalic prosomeres (hp1/hp2); a thicker red dash line marks the diencephalo-hypothalamic boundary (PTh/hp1; diencephalic Th and PTh are indicated without boundaries). The intrahypothalamic limit separates the peduncular and terminal hypothalamic portions (PHy, THy). The hypothalamic and preoptic acroterminal region is demarcated with a green dash line. The prominent anterior commissure identifies the rostral end of the forebrain roof plate, whereas the floor plate ends under the mamillary body (Mm). The rostral part of the infundibular region (In), and the cell groups identified as dorsal infundibular (DIn) and alar subventricular (Sv) nuclei (probably jointly representing the suprachiasmatic nucleus) form part of the basal and alar hypothalamic acroterminal region, respectively. The preoptic part of the acroterminal region contains the terminal lamina (LT), and the paramedian preoptic nucleus, identified here as the periventricular nucleus (Pep), ending at the anterior commissure (ac). In the basal hypothalamus, the large THy comprises mamillary and tuberal formations such as the mamillary nucleus (Mm), and the dorsomedial (Dm), dorsal premamillary (DP) and ventromedial (Vm) nuclei, the tuber cinereum area (TCA), and part of the infundibular or arcuate nucleus (In); the perimamillary area (Pem) corresponds to the mamillary floor. The basal PHy contains the posterior hypothalamic area (PA) and the supramamillary nucleus (Sm; our retromamillary nucleus or RM). In the alar hypothalamus, there clearly appears the dorsoventral subdivision into the paraventricular area (with dorsal and anterior portions -Pd, Pa- corresponding to the peduncular and terminal subregions) and subparaventricular area (SPa; including portions identified as the alar nucleus and anterior hypothalamic area, Al, AA). Reprinted by permission of the University of Wisconsin Press. © 1985 by the Board of Regents of the University of Wisconsin System. All rights reserved.

There is otherwise various data on the developing human hypothalamus in Müller and O’Rahilly (1988, 1989a,b, 1990a,b) and O’Rahilly and Müller (1999) or the adult and embryonic primate hypothalamus in Bleier (1984); see Figure 5 and Gribnau and Geijsberts (1985). More recently, Koutcherov et al. (2003) analyzed chemoarchitectonically the developing human hypothalamus through fetal and postnatal stages in coronal sections. These authors offered a classical columnar interpretation of hypothalamic structure, simply dividing it into midline (periventricular), core, and lateral (superficial) zones.

## Morphogenetic Organizer Centers of the Hypothalamus

Like the rest of developing brain regions, the prospective forebrain containing the future hypothalamus is under the influence of multiple diffusible signaling molecules produced by extraneural (primary) and neural (secondary) organizers (reviewed in Echevarría et al., 2003; Vieira et al., 2010; Anderson and Stern, 2016; Puelles, 2017). These provide inductive (instructive, repressive) or positional signals which contribute to progressive regionalization, defining more precisely the neural identity or molecular profile of the diverse territories that fall within the range of these organizer systems. Their joint action triggers finer dorsoventral and anteroposterior regionalization in specific neuroepithelial progenitor subdomains. In general, the primary organizers are the main inducers of *neural* character or fate, a mechanism thought to occur by repression of previously inbuilt ectodermal specification as *epidermis* (Holley et al., 1995; Wilson and Hemmati-Brivanlou, 1995; De Robertis and Sasai, 1996). Primary neural induction is largely “vertical” since it involves signals coming from a different embryonic layer, i.e., axial mesoderm or endoderm, which act upon the ectoderm (reviewed in Doniach, 1993; Ruiz i Altaba, 1994; Echevarría et al., 2003; Vieira et al., 2010). Contrarily, secondary organizers develop as specializations of the early neuroepithelium, and their signals act mainly by diffusion or propagation by cell-cell contact in the plane of the neural wall (i.e., “planar” effects). Secondary organizers are largely responsible for brain wall differential regionalization (progressive subdivision and delimitation into differentiated smaller neuroepithelial areas; reviewed in Stern, 2001; Echevarría et al., 2003; Puelles, 2017). Positional information is provided by signaling diffusible morphogen molecules such as Sonic Hedgehog and Fibroblast Growth Factors (SHH, FGFs) are secreted by specific secondary organizers and diffuse gradientally. Nearby portions of the neuroepithelium sensible to these molecules react variously depending on the concentration sensed (different according to the distance from the morphogen source), essentially triggering selectively one of the various genetic signalings cascades possible at each locus (Wolpert, 1969, 2016; Gurdon and Bourillot, 2001; Cohen et al., 2013). The newly activated genes modulate the function of pre-existing regulatory genes such as transcription factors or induce the expression of new ones, including differentiation genes (Shimamura et al., 1995; Crossley et al., 1996; Martinez et al., 1999; Martínez, 2001; Echevarría et al., 2003; Davidson, 2006; Puelles and Ferran, 2012; Anderson and Stern, 2016).

The effect of *different* emerging molecular cascades driven by characteristic *combinations* of transcription factors occurring selectively in given parts of a neural territory leads to its subdivision into as many parts as those that develop a variant molecular profile, with their now distinct boundaries, potencies, and *fates* (Echevarría et al., 2003). The earliest molecular boundaries formed at neural plate stages are quite dynamic, often adjusting and refining rapidly their relative position and limits over time, due to agonistic or antagonistic interactions with gene products and intercellular signals produced at neighboring areas (Sánchez-Arrones et al., 2009, 2012). However, this dynamism diminishes or disappears once the neural tube closes, so that boundaries thereafter tend to be persistent, and novel ones simply subdivide former larger fields of neuroepithelium into smaller domains. We assume for simplicity that normally all matrix cells contained within a specific molecularly delimited area express the same set of genes, thus allowing for an “areal” collective manifestation of the same properties and effects within the limits of the area. The area possesses a degree of *fate specification*, or manifests a variable or permanent *state of determination*; it behaves as a polyclonal unit with shared molecular properties as long as it does not suffer subdivision by further patterning effects. Adult areal units reach a nearly permanent state of determination. This systematic and progressive molecular compartmentation process is known as “regionalization.” The latter is first molecular (genes active or repressed in the neuroepithelial cells, with the corresponding incipient molecular boundaries), and leads to the area carrying distinct developmental *potencies*. Later the different molecular profiles start to affect differentially *via* the corresponding genetic cascades both local proliferation and neurogenesis (birth of neurons) and ulterior differentiation, leading eventually to histologically and anatomically *visible* boundaries and regions of the brain (Puelles and Ferran, 2012; Nieuwenhuys and Puelles, 2016).

This theory can explain how a specific brain nucleus or a set of nuclei form in typical relative positions. Such explanations were not possible in the non-molecular era of neurobiology. First, a neuroepithelial tissue with generic neural properties arises *via* vertical signaling (primary organizer signals, neural plate, and neural tube). Next, various secondary organizers emerge at specific sites and start to release gradientally their signals. Some vertical signals may continue active. Some antagonistic effects may occur between adjacent areas expressing incompatible molecular profiles, each one tending to annulate or change the borders of the other. This dynamic preliminary neural plate stage leads in the closed neural tube to a provisional and gradually changing equilibrium of brain wall regionalization, creating finer molecularly distinct microzones or progenitor areas. Each set of distinct matrix cells lose many of their initial potencies, becoming finally restricted to producing only given neuronal types, due to the distinct constellation of genes they keep active. Once the resulting neuronal and glial derivatives are set in place and differentiate in the mantle layer, we have the incipient nucleus. Some nuclei, or most complex structures, such as cortical and reticular formations, resulting from the aggregation and functional interaction of mixtures of neuronal types produced in different progenitor areas.

**Figure 6:**
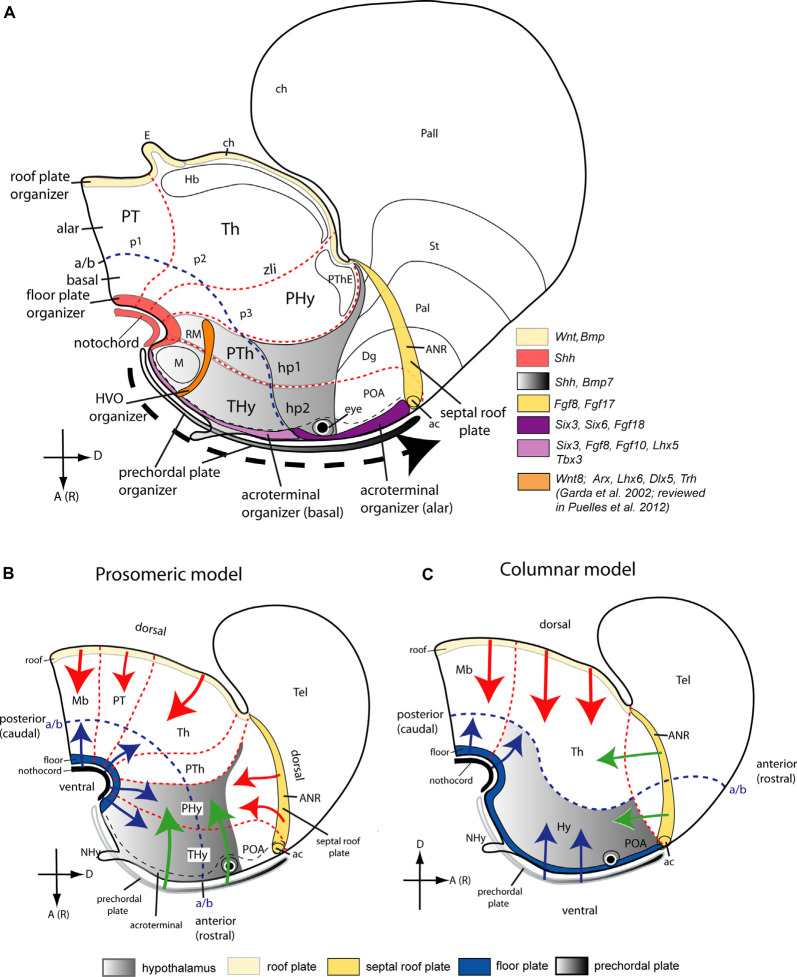
Topological location of seven postulated morphogenetic organizers thought to influence regionalization of the hypothalamus, represented upon the prosomeric model **(A)**, and in complementary diagrams **(B,C)** that compare dorsalizing, ventralizing, and rostralizing patterning effects theoretically acting on the hypothalamus *via* signals diffusing from the roof, the notochord or the floor, or the prechordal plate, as they would be differentially conceived in the updated prosomeric model **(B)** vs. the columnar model **(C)**. Schemata represent the forebrain of mouse embryos at approximately E15.5 **(A)** and E13.5 **(B,C)**. The interprosomeric borders are identified as red dash lines and the alar/basal limit as a blue dash line. Anterior [or rostral; A(R)] and dorsal (D) spatial hypothalamus dimensions are indicated in **(A)**. **(A)** Color-coded-map of various genes expressed in seven potential morphogenetic organizers of the hypothalamus (modified from Puelles, 2017): prechordal plate (gray gradient and black dash arrow indicating the dorsalward prechordal cell migration in front of the acroterminal area (AT) from the floor neighborhood to the roof), anterior neural ridge roof plate (ANR, yellow), notochord and floor plate (light red), basal and alar acroterminal regions (lavender and violet, respectively) and hypothalamic ventricular organ (HVO, orange). The hypothalamic area displays a dorsoventral grey gradient. Transverse prosomeric units are numerically identified: p1, p2, and p3, and their corresponding pretectal, thalamic, and prethalamic alar subdomains in the diencephalon, and hypothalamo-telencephalic prosomeres (hp1, hp2). The hp1 segment contains the peduncular hypothalamus (PHy) plus the evaginated telencephalon (pallium, Pall, and subpallium -striatal, pallidal, and diagonal regions, St, Pal, Dg). The hp2 segment comprises the terminal hypothalamus (THy) plus the preoptic region (POA), and also contains the AT (unless the latter is considered a singular median hp3). Some other landmarks are also identified: anterior commissure (ac); habenula (Hb), mamillary and retromamillary regions (M, RM); prethalamic eminence (PThE). **(B,C)** Comparison of rostralizing (green arrows), ventralizing (blue arrows), and dorsalizing (red arrows; patterning effects either in the prosomeric model or in Swanson’s columnar model modified from Puelles and Rubenstein, 2015). The hypothalamus is identified with a gradiental grey background. Note the radical difference in the conception of the alar/basal boundary (defining the length axis). The hypothalamus lies rostral to the diencephalon **(B)**vs. ventral to it **(C)**. The different concepts about the anteroposterior (or rostrocaudal) and dorsoventral spatial dimensions in these models alter drastically the interpretation of the patterning effects of the organizers. In the prosomeric model **(B)**, an anteroposterior subdivision of the hypothalamus into the terminal and peduncular parts (THy, PHy) is interpreted as the effects of the prechordal plate and perhaps also the acroterminal area (green arrows), mediated mainly by SHH, NODAL and FGF morphogens. Diffusible molecules such as SHH spread from the notochord the hypothalamic floor plate, and (later) the basal plate, with ventralizing effects (blue arrows) antagonistic to dorsalizing effects of signals released from the ANR in the roof plate (e.g., FGF8; red arrows). Consequently, the antagonism between floor and roof dorsoventral patterning effects may produce the alar/basal border, as well as the tel-hypothalamic border, the basal mamillary/tuberal subdivisions, and the alar paraventricular/subparaventricular areal subdivisions. In contrast, as schematized in **(C)**, Swanson’s columnar model, by postulating an extended “rostral floor plate” over the prechordal plate (blue arrows) implicitly tends to interpret the hypothalamus as a diencephalic basal plate extending into telencephalic “basal” ganglia but loses any possibility to explain the mamillary/tuberal and paraventricular/subparaventricular subdivisions, since they become anteroposterior differentiations within the basal domain. There is no recorded theory about why the columnar hypothalamus divides into mamillary, tuberal, anterior, and preoptic domains. Similarly, the ANR signals have to be interpreted (and have indeed been interpreted so in the literature) as a *rostralizing* influence (green arrows), though they come indisputably from the preoptic *roof plate* Abbreviations: ac, anterior commissure; Mb, midbrain; NHy, neurohypophysis; POA, preoptic area; PT, pretectum; PTh, prethalamus; Tel, telencephalon.

Any alteration of the regionalization process may produce abnormalities in the final functional cellular structure of the brain (missing, quantitatively abnormal, or badly placed sets of neurons), with consequent effects on the emergent functions. The combined effect of signaling molecules from different organizing sources thus progressively divides the neuroepithelial wall into domains and subdomains with differential genetic patterns. This produces the complex neuronal architecture of the mature brain. The causal explanation requires identifying and following the effects of the relevant organizers in normal and pathological conditions. However, the specific organizer effects operating in the prospective hypothalamic territory are so far only sparsely known. We will summarize the somewhat confused state of the literature on this topic (largely due to the shift in morphological models) by commenting on seven apparent sources of diffusible signaling molecules. These represent more or less established candidate morphogenetic organizers acting upon the prospective hypothalamus: (1) the prechordal plate (rostralizing), and (2) the notochord (ventralizing), a primary vertical action organizer; (3) the floor and basal plate (secondary ventralizing), (4) the anterior neural ridge (ANR) or future roof plate (dorsalizing), (5/6) the median alar and basal acroterminal region (secondary rostralizing), and (7) the hypothalamic ventricular organ (Figure 6A).

### Prechordal Plate

Fate-mapping and ablation studies suggest that the *prechordal plate* is an extraneural organizer acting rostrocaudally—AP dimension—upon the rostral forebrain (a moving organizer, since its cells migrate ventrodorsally in front of the AT). It participates dynamically in early AP organization of the prospective hypothalamus and has a substantial role in the bilateral separation of the eye and telencephalon fields (Li et al., 1997; Pera and Kessel, 1997; Shimamura and Rubenstein, 1997; Camus et al., 2000; Kinder et al., 2001; García-Calero et al., 2008; Aoto et al., 2009). At early primitive streak stages (early gastrulation), the prechordal mesodermal cells originate from the anterior end of the node and migrate dorsalward along the overlying median rostral terminal wall of the neural plate (prospective acroterminal region; Figure 6; e.g., Kinder et al., 2001). Later, they reach the rostral end of the neural tube roof plate, where the anterior commissure forms (Figure 6A, dotted arrow). If the entire forebrain is epichordal (i.e., has a floor plate and underlying notochord), as is proposed in the updated prosomeric model, the prechordal cells move ventrodorsally concerning the topology of the closed neural tube (columnar authors wrongly hold that it extends *rostralwards under* the hypothalamus; Figures 6B,C). In contrast with the DV ventralizing role of the notochord, the advancing prechordal plate cell population exerts over time a series of AP-patterning effects upon all the primary longitudinal zones of the rostralmost forebrain, present at the prospective AT (compare Figures 6B,C; Puelles and Rubenstein, 2015; Puelles, 2017). Alternative columnar interpretations of conjectural prechordal “DV signaling” on prospective hypothalamus are available (Bedont et al., 2015; Figure 1; Xie and Dorsky, 2017; Figure 2).

Along their para-acroterminal migratory route, the signals secreted by the prechordal cells (e.g., SHH, BMP7; Dale et al., 1997, 1999; Manning et al., 2006; García-Calero et al., 2008) would first promote specification of the mamillary body and enlargement of the tuberal hypothalamus, including the infundibulum and neurohypophysis (basal plate effects). Thereafter they contribute to separate formation of right and left eyes from the median eye field (by repressing this fate at the midline), and finally similarly induce the separation of the two telencephalic hemispheres, inducing also the preoptic terminal lamina (alar plate effects). The sequential removal of the prechordal plate at different times during the gastrulation period produces a range of holoprosencephalic phenotypes (García-Calero et al., 2008). Late ablations produce a nearly normal forebrain, while removal of the prechordal plate at the earliest primitive streak stages cause the largest forebrain defects, comprising absence of the basal hypothalamus, cyclopia, and undivided telencephalic hemispheres, similarly to malformations observed in *Shh*-defective homozygotic mutants (Chiang et al., 1996; García-Calero et al., 2008). Moreover, early removal also causes underdevelopment or complete absence of basal and floor components of the diencephalon and midbrain (García-Calero et al., 2008). This suggests that the signaling range of the prechordal cells includes these prospective territories at the earliest gastrulation stages, in apparently necessary interaction with notochordal signals (smaller distances all around). In the rat, ablation of prechordal cells also generates diverse holoprosencephalic phenotypes apparent at E9 (Aoto et al., 2009); the surgical ablations were performed at a presomitic stage (zero-somite stage; equivalent to E7.75 in mice) apparently between mid to late primitive streak stages. However, the range of mild to severe phenotypes found by these authors was ascribed to heterochronic aspects of prechordal plate formation in littermates (Theiler, 1989; Fujinaga et al., 1992; Downs and Davies, 1993).

### Floor Plate

The *floor plate* is conventionally recognized as a ventralizing secondary organizer formed at the ventral midline of the neural tube at the prospective spinal cord, hindbrain, and “midbrain” territories; note the latter often included what we now interpret as diencephalic and even hypothalamic floor areas (Figure 6B; Placzek et al., 1991; Yamada et al., 1991; Roelink et al., 1994; Sasaki and Hogan, 1994; Ericson et al., 1995a; Hynes et al., 1995). Conventionally, a similar inductive role of the floor plate at forebrain diencephalic and hypothalamic levels is often unrecognized, due to the classic wrong assumption that the notochord, as well as the floor plate, are absent at these territories (Kingsbury, 1930; Ericson et al., 1995a; e.g., Placzek and Briscoe, 2005 place the notochordal rostral end at the interthalamic zona limitans; their Figure 1B). Several studies identify instead, the prechordal SHH source as the earliest *ventralizing* organizer acting upon the prospective forebrain (Figure 6C; Yamada et al., 1991; Echelard et al., 1993; Shimamura et al., 1994; Dale et al., 1997; Shimamura and Rubenstein, 1997; reviewed in Placzek and Briscoe, 2005; see their Figure 1). The range of this hypothetic effect would include particularly the entire hypothalamus. This is obviously a notion derived from the supposed longitudinal nature of the hypothalamus “column” within the columnar forebrain model, which disregards the axial role of the notochord. Recent molecular and experimental evidence supporting the prosomeric model contradicts this interpretation (e.g., floor and basal *Shh*, alar-basal *Nkx2*.2, alar Pax6 expression patterns; *Six3* loss of function phenotype described by Lagutin et al., 2003).

The floor plate is the most ventral longitudinal zone of the neural tube, and the prosomeric model explicitly defines it throughout the neural tube. The expression pattern of several genes circumscribed to the forebrain floor plate supports that the floor region ends at the hypothalamic mamillary pouch, coinciding with the primary rostral end of the underlying notochord (Puelles et al., 2012; Puelles and Rubenstein, 2015). The direct inductive apposition between both structures is only transiently visible at very early embryonic stages (Figure 6A; Puelles and Rubenstein, 2015; their Figure 11). Substantial data support that the prosomeric diencephalic and hypothalamic floor plate differentiates as an axial SHH effect produced by the *primary* *notochordal organizer* (Figure 6B; Bovolenta and Dodd, 1991; Placzek et al., 1993, 2000; Ruiz i Altaba et al., 1993; Roelink et al., 1994; Ericson et al., 1995b; Martí et al., 1995; Dale et al., 1999; Placzek and Briscoe, 2005). The hypothalamic floor (as all other forebrain floor portions) activates itself the *Shh* gene from neural plate stages onward, and homeotically induces subsequently *Shh* expression at the local hp1 basal plate (Figure 6A; Echelard et al., 1993; Roelink et al., 1994; Ericson et al., 1995a; Shimamura et al., 1995; Placzek and Briscoe, 2005; Aoto et al., 2009). Importantly, only the floor and basal portions of the acroterminal domain (either a part of hp2 or hp3) express primarily *Shh*. The prechordal plate ventralization hypothesis would wrongly predict the same result at the alar AT, since all the hypothalamus is underlined by the prechordal plate, and what we consider *alar AT* is held to be floor/basal in the columnar model. Therefore, we consider the retromamillary (hp1) and mamillary (hp2) hypothalamic floor plate (amplified secondarily by the basal plate) as a hypothalamic organizer source of secreted SHH signal which is shared qualitatively by the whole forebrain. This participates in the DV differentiation of the hypothalamic alar and basal plates, as well as in the positioning of the *Nkx2.2*-positive alar-basal boundary (Figure 1C; Puelles and Rubenstein, 2015).

This does not rule out an early inductive effect of prechordal plate cells on the prospective mesencephalic, diencephalic, and hypothalamic basal plate (García-Calero et al., 2008). The expression of *Nkx2.1*, a characteristic marker of the basal hypothalamus, was absent following the removal of the prechordal plate in mouse forebrain neural plate explants at 0–1-somite stages (Shimamura and Rubenstein, 1997). However, basal hypothalamic *Nkx2.1* expression emerged when extirpations were carried out at later stages, probably because the migratory prechordal cells were no longer in contact with the hypothalamic basal acroterminal region, having advanced to the alar acroterminal region at these stages (Shimamura and Rubenstein, 1997).

A potential summation effect of SHH inductive signals coming from the floor plate, basal plate, and prechordal plate, probably mediated by a particular enhancer of the SHH signaling pathway (Jeong and Epstein, 2003; Lee et al., 2012), is the ventrodorsal expansion of the basal hypothalamus (e.g., Figures 3, 6). This might explain the great expansion of the hypothalamic basal plate compared to neighboring diencephalic tegmental domains.

### Anterior Neural Ridge

The *ANR* is a putative secondary organizing center of the telencephalon and hypothalamus. We refer to the bilateral neuroectodermal ridge that limits rostrally the neural plate (note there is no neural crest at this level). The ANR receives vertical FGF8 and other primary signals from the underlying anterior visceral endoderm, crucial for the development of the telencephalon (Sánchez-Arrones et al., 2012). As an embryonic structure, it is present from early neural plate stages until the closure of the anterior neuropore, when it transforms into the hypothalamo-telencephalic roof plate, but we do not know when it starts releasing morphogens that pattern the rostral forebrain (ANR; Figure 6A). The literature generally identifies the ANR within columnar assumptions as an AP organizer of the rostralmost prosencephalon (Houart et al., 2002; Lagutin et al., 2003; reviewed in Echevarría et al., 2003; Vieira et al., 2010). However, the ANR lies at the border of rostral neuroectoderm with non-neural ectoderm (Eagleson et al., 1995; Shimamura and Rubenstein, 1997; Houart et al., 1998; Sánchez-Arrones et al., 2009, 2012), and its fused neural material later represents the median *septo-commissural* roof plate, that is the telencephalo-hypothalamic roof plate (Figure 6A; Cobos et al., 2001). Accordingly, it should function from the beginning as a *dorsalizing* *organizer*, like all other roof-plate signaling centers in the neural tube, and the rest of the neural/non-neural border in the neural plate, excluding the neural crest. We accordingly postulate within the prosomeric model that the ANR organizer has a dorsalizing role both at long range relative to the acroterminal domain and neighboring terminal and peduncular hypothalamic regions, and at close range, upon the associated non-evaginated and evaginated parts of the telencephalon (Beccari et al., 2013; Puelles and Rubenstein, 2015; Puelles, 2017, 2019). Indirectly, its dorsalizing effects upon the AT domain (e.g., the latter’s division into alar and basal moieties, or the early neural plate expression domains of Six3 and Hesx1; Lagutin et al., 2003) may modulate its secondary AP patterning effects (see below). Apart of its dorsalizing effects upon the prospective telencephalon and alar hypothalamus it possibly influences as well early on the fate of the eye fields (see Figures 1A, 6C; e.g., Crossley et al., 2001; Xie and Dorsky, 2017). Thus, the ANR effects antagonize the ventralizing SHH signal secreted by the *floor plate* at the hypothalamic mamillary/retromamillary levels (ANR, M, RM, floor plate organizer; Figure 6B).

The ANR expresses *Fgf* family members such as *Fgf8* and *Fgf17* at neural plate stages in mouse and chick embryos (Crossley and Martin, 1995; Shimamura and Rubenstein, 1997; Maruoka et al., 1998; Crossley et al., 2001; Cholfin and Rubenstein, 2007; Sánchez-Arrones et al., 2009; Cajal et al., 2012; Sánchez-Arrones et al., 2012). Various experimental work, including studies on *Fgf8* hypomorphs, shows that the ANR has regulatory FGF8-mediated effects on the anterior forebrain (Shimamura and Rubenstein, 1997; Houart et al., 1998; Ye et al., 1998; Heisenberg et al., 1999; Crossley et al., 2001; Fukuchi-Shimogori and Grove, 2001; Storm et al., 2003, 2006; Paek et al., 2009). However only the knockout of both *Fgf8* and *Fgf17* genes leads to rostral forebrain defects, the phenotype being milder in *Fgf17* knockout mice (Garel et al., 2003; Storm et al., 2006; Cholfin and Rubenstein, 2007, 2008).

### Acroterminal Domain

The hypothalamus presents along its acroterminal domain (AT) at least two postulated AP dimension organizers (alar and basal AT of Puelles, 2017), possibly set in place under prechordal, anterior visceral endoderm and ANR influence. The alar and basal parts of the AT express genes coding for SHH receptors (e.g., LRP2, Christ et al., 2012), *Shh* response elements (*Gli3*, Haddad-Tóvolli et al., 2015), SHH repressors (*Tbx2/3*, Manning et al., 2006; Pontecorvi et al., 2008), diffusible morphogens of the *Fgf* and *Bmp* families (Manning et al., 2006; Ferran et al., 2015; Fu et al., 2017; Placzek et al., 2020). These are differently combined at the alar and basal parts of the acroterminal region, though the accrued data suggest further DV regionalization of the AT; some data commented below suggest distinct acroterminal mamillary and acroterminal tuberal organizers (Figures 6A,B; Ferran et al., 2015; Puelles and Rubenstein, 2015; Puelles, 2017). The *anteroposterior* (AP) patterning effects are interpreted as affecting the DV dimension of the hypothalamus within the columnar tradition (e.g., Kapsimali et al., 2004). There is also a novel hybrid model proposed by Placzek and collaborators (Fu et al., 2019; Placzek et al., 2020).

*Fgf18*, which is expressed in the *alar acroterminal* subdivision (Figure 6A; Ferran et al., 2015) may have a rostralizing role upon the POA (where an SHH-releasing subpallium organizer emerges; Puelles, 2017) and the underlying alar hypothalamus, including potential effects on the prospective eye field within the early AT, and the paraventricular, supraoptic and suprachiasmatic nuclei subsequently. This hypothesis has not been explored so far experimentally.

On the other hand, the entire alar portion and the tuberal basal part of the acroterminal region (i.e., excepting the mamillary subregion) express *Six3* from early neural plate stages onwards (Ferran et al., 2015; Yu et al., 2017). This gene was found to have an antagonizing (*rostralizing*) effect against caudalizing WNT signals found more caudally in the forebrain (Lagutin et al., 2003). *Six3* is one of the genes altered in holoprosencephaly (Schell et al., 1996; Solomon et al., 2009). However, it remains unclear how *Six3* counteracts WNT signals since it is a transcription factor. One possibility is that it activates *secreted frizzled-related peptide* (*Sfrp*) genes, whose secreted products block WNTs attachment to the receptor Frizzled (see below for *Lhx5*).

Moreover, Haddad-Tóvolli et al. (2015) studied experimentally the role of *Gli1*, *Gli2*, and *Gli3* as SHH response elements in the patterning of the hypothalamus, using as reference the prosomeric model. They observed that alar hypothalamic and preoptic regions did not require *Gli2* or *Gli3* genes, but did depend strongly on prechordal SHH and neural-expressed SHH (probably from the local preoptic source). The basal hypothalamus (particularly its acroterminal and terminal parts; THy) depended importantly on early Gli2A activation at E7.0 by exogenous SHH (prechordal and/or notochordal). Lack of this function caused a *caudalized* phenotype, in which acroterminal-derived structures (median eminence, neurohypophysis) essentially disappeared, whereas tuberal nuclei, such as the ventromedial nucleus and the arcuate nucleus, appeared fused into unpaired masses in place of the corresponding basal AT. This reveals that the formation of the AT itself requires SHH induction mediated by *Gli2*, probably starting from prechordal plate signals, known to have a selective *Shh* enhancer (Lee et al., 2012; Sagai et al., 2019). The gene *Gli3* only had a role in the development of the mamillary body, influencing its proliferation. The lateral hypothalamus (PHy) depended mainly on a neurally-expressed SHH signal, which at this level probably results from the local floor and basal plate, which both express *Shh*.

**Figure 7:**
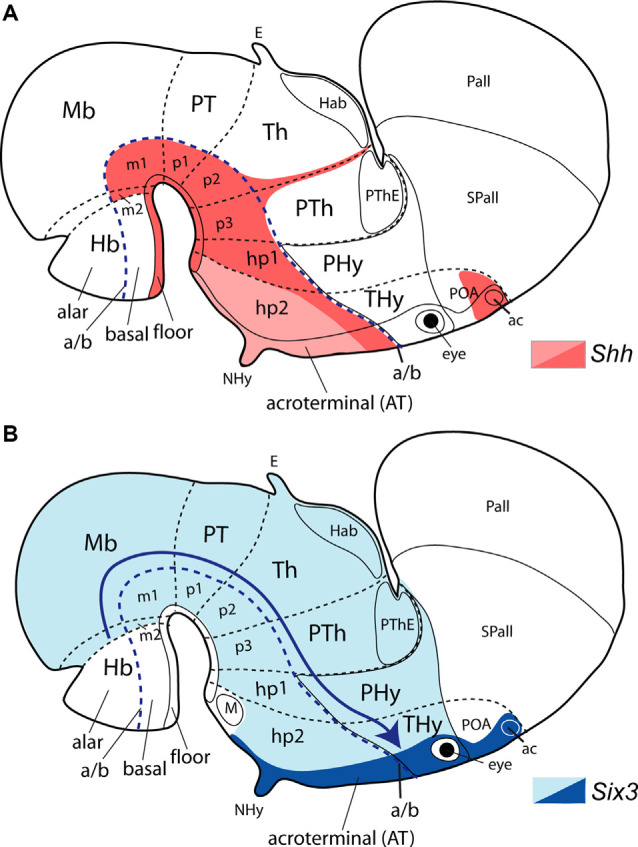
Schematic maps of the dynamic expression of *Shh* and *Six3* represented upon the prosomeric model. All forebrain prosomeres are identified; midbrain mesomeres (m1 and m2), diencephalic prosomeres [(p1, p2, p3 and their respective pretectal, thalamic and prethalamic alar components (PT, Th, PTh)] and secondary prosencephalic or hypothalamo-telencephalic prosomeres (hp1 and hp2, eventually hp3 as acroterminal region, AT). Note hp1 contains the peduncular hypothalamus (PHy) and evaginated telencephalon (Pallium, Pall; subpallium, Spall), whereas hp2 includes the terminal hypothalamus (THy) and the unevaginated telencephalic POA. The eye vesicles fall within the AT territory. **(A)** *Shh* is expressed early on, from neural plate stages onwards, in the forebrain floor and basal plates, in contrast to only at the floor plate in the hindbrain (red background) This is secondarily downregulated in a large median mamillo-tuberal region by repressive BMP-TBR2 signals (pink). A later-appearing separate *Shh*-expressing locus emerges at the preoptic region (POA; red). **(B)** *Six3* quickly expands early on at neural plate stages from the acroterminal region backward, down to the midbrain/hindbrain border (isthmic limit; light blue area). Later, at neural tube stages, this large domain is progressively downregulated (blue arrow) caudally by interaction with repressive *Irx* gene function, so that *Six3* results finally reduced in its expression mainly to the acroterminal region (dark blue), excepting its mamillary portion (white). Ulteriorly, *Six3*-expressing cell derivatives appear at the prethalamic reticular nucleus, and in amygdalar and septal regions (Sánchez-Arrones et al., 2009; Dutra de Oliveira Melo, 2011). Other abbreviations: ac, anterior commissure; E, epiphysis; Hab, habenula; Hb, hindbrain; M, mamillary region; Mb, midbrain; NHy, neurohypophysis; PThE, prethalamic eminence.

There is no basal AT expression of *Six3* at the tubero-mamillary transitional area and the mamillary/retromamillary basal subregions (ventralmost basal plate; Figure 7B). In contrast, *Foxb1, Lhx1, Sim1/2*, and *Lhx5* are selectively expressed early on at the mamillary portion of the AT and neighboring mamillary area, and these genes are required for normal mamillary body development (Wehr et al., 1997; Alvarez-Bolado et al., 2000; Radyushkin et al., 2005; Heide et al., 2015). In zebrafish, *Lhx5* inhibits caudalizing *Wnt* genes by acting upstream of the *Wnt* antagonists *Sfrp1a* and *Sfrp5* (Peng and Westerfield, 2006), which implies it exerts a *rostralizing* effect. The distinction between retromamillary and mamillary areas likely results from WNT vs. *Lhx5* antagonism (Heide et al., 2015).

The SHH repressor gene *Tbx2/3* appears expressed selectively in the chick (*Tbx2*) and mouse (*Tbx3*) tuberal region, inciding somewhat into the mamillary area (Manning et al., 2006; Pontecorvi et al., 2008). Its expression depends likewise upon *Lhx5* function at the mamillary AT subdomain, and other genes involved at this locus including *Foxb1, Lhx1*, and *Bmps* (Heide et al., 2015; Fu et al., 2019). The potential mouse *basal acroterminal organizer* thus expresses differentially *Tbx3*, *Lhx5*, *Fgf10*, and *Fgf8* (Figure 6A; Crossley and Martin, 1995; Rubenstein et al., 1998; Parkinson et al., 2010; Ferran et al., 2015; Fu et al., 2017; Allen Developing Mouse Brain Atlas). Mouse *Tbx3* (similarly as *Tbx2* in the chick) correlates in expression with the tuberal and tuberomammillary part of median basal AT territory that initially expresses *Shh* presumably under dual prechordal and floor plate induction, but where this signal is repressed subsequently by adenohypophysial signals and *Tbx3*-BMP action (Manning et al., 2006). This repression does not eliminate *Shh* expression next to the alar-basal boundary. The repressed AT and THy basal tuberal area is important for patterning of the tuberal infundibulum, median eminence, arcuate nucleus, and neurohypophysis (Manning et al., 2006; Trowe et al., 2013; Heide et al., 2015; Fu et al., 2019). Finally, FGF8/10 signals diffused caudalwards from the basal acroterminal subdomain probably participate in additional rostralizing anti-WNT effects in the prospective basal hypothalamus, probably mainly at its tuberal/retrotuberal domains. The literature usually attributes confusingly such effects to the more distant FGF8 source at the ANR, whose effects are dorsoventral rather than anteroposterior (see references cited above; Figure 6; compare b and c).

### Hypothalamic Ventricular Organ

Finally, the *hypothalamic ventricular organ* (HVO, or paraventricular organ, HPV) is a linear longitudinal ependymal specialization running across basal PHy and THy (Figure 6A), which is well-known in non-mammalian vertebrates and is not generally described in mammals (but see Puelles et al., 2012). Nowadays its identification is easier even in mammals by the fact that its ependymal cells express intensely *Wnt8b* at early embryonic stages, as observed at least in the chick and mouse (Garda et al., 2002; Bardet, 2007). Data presented in recent publications suggest that the HVO appears related topographically to different molecularly defined neuronal populations in the surrounding mantle layer across PHy and THy (Puelles et al., 2012; Li et al., 2018; Diniz et al., 2019). WNT8 is one of the diffusible members of the WNT family and is also relevant for thalamic patterning (Puelles and Martinez, 2013). This longitudinal basal organizer appears dorsal to the floor plate and ventral to the alar-basal boundary, coursing parallel to these two landmarks. It lies along the boundary between the major tuberal/retrotuberal areas and the perimamillary/periretromamillary areas (actually it represents the ependyma of the thin ventral Tu/RTu subdomain, where histaminergic neurons are produced; Puelles et al., 2012). This location confers to it an unexplored DV modulating role inside the basal hypothalamus and its tuberal and mamillary specializations, mediated apparently by the WNT8 morphogen (HVO; Figures 6A,B; Puelles et al., 2012; Puelles, 2017).

## Alterations of Genes Implicated in Hypothalamic Development Underlie Human Forebrain Congenital Abnormalities

Next, we will focus on some molecules produced by organizers of the prospective hypothalamus whose abnormal nature (mutation) or distribution may underlie anomalies in the development of the human forebrain. Indeed, mutations in signaling molecules (e.g., SHH and FGFs) and transcription factors (e.g., SIX3, SOX2, and SOX3) have been implicated in midline forebrain defects such as septo-optic dysplasia and holoprosencephaly. However, only 25% of holoprosencephalic cases are due to mutations in known genes correlated with holoprosencephaly (e.g., *Shh, Fgf8, Six3*), leaving 75% of cases with unknown mutations (Dubourg et al., 2004, 2018; Pineda-Alvarez et al., 2010). In septo-optic dysplasia there are few family cases, the majority of cases being sporadic; thus, less than 1% of septo-optic dysplasia patients have mutations in the genes *Hesx1, Sox2, Sox3*, or* Otx2* (Webb and Dattani, 2010). Both rare congenital diseases are heterogeneous genetic and clinical disorders. Septo-optic dysplasia in Europe has an incidence of 1.9–2.5 cases per 10,000 births (Garne et al., 2018), while the prevalence of holoprosencephaly is 1 case in 10,000 births (Leoncini et al., 2008; Kauvar and Muenke, 2010; Yi et al., 2019; see a systematic review in Orioli and Castilla, 2010).

In humans, holoprosencephaly is the most common developmental anomaly of the forebrain and face. It is characterized by a spectrum of abnormalities ranging from extreme malformations displaying a single undivided forebrain, cyclopia, and proboscis formation (alobar holoprosencephaly, sometimes accompanied by complete dorsalization of the entire forebrain, i.e., no basal plate) to mild alterations such as the absence of the corpus callosum, arrhinencephaly and hypotelorism (lobar holoprosencephaly; reviewed in Cohen, 2006; Dubourg et al., 2007). Lack of a forebrain basal plate by complete dorsalization is the pattern observed by García-Calero et al. (2008) in chick embryos whose prechordal plate was removed experimentally. Mutations in developmental genes implicated in the SHH, FGF, or TGF signaling pathways contribute to holoprosencephaly (reviewed extensively in Roessler and Muenke, 2010; Roessler et al., 2018). Environmental factors such as hyperglycemia and exposure to retinoids or ethanol increase the risk of holoprosencephaly, due to their teratogenic effects on prechordal cell migration and their signaling molecules (Blader and Strähle, 1998; Cohen and Shiota, 2002; Aoto et al., 2008; Miller et al., 2010; Hong and Krauss, 2012, 2017; Kietzman et al., 2014; Billington et al., 2015).

### *Shh*/SHH

*Shh* is one of the main genes altered in holoprosencephaly, SHH-haploinsufficiency being relatively frequent in patients (Roessler et al., 1996; Nanni et al., 1999; Mercier et al., 2011; Dubourg et al., 2016). Mutations in this gene are associated with 17% of holoprosencephalic familial cases and 3.7% of sporadic cases (Cohen, 2006). However, members of the same family carrying identical *Shh* mutations can display a great variability of mild-to-severe holoprosencephalic phenotypes. As was described previously, the SHH signaling molecule is produced at several organizer centers (notochord, prechordal plate, floor plate, basal plate, POA), some of which act nearly simultaneously, and others at different developmental times, on the whole, forebrain primordium, or on particular areas such as the prospective hypothalamus (Figure 6). Thus, experimental blocking of SHH signaling at different developmental times by exposing chicken embryos to different doses of the steroidal alkaloid cyclopamine generates a range of craniofacial and brain malformations correlated with temporal disruption of various *Shh* functions (Cordero et al., 2004; Mercier et al., 2013).

Exposure to ethanol at different gestational periods produces defects related to either holoprosencephaly or septo-optic dysplasia (Lipinski and Bushman, 2010; Lipinski et al., 2010; Kietzman et al., 2014). In any case, the most severe malformations occur after early ablation of *Shh*-expressing prechordal cells, or blockage of signals released from the median prechordal cells (Li et al., 1997; Pera and Kessel, 1997; Wallis and Muenke, 2000; Aoto et al., 2009). The cited experiments reproduce the severe holoprosencephalic phenotype of the *Shh*-null homozygous mice (Chiang et al., 1996). The more subtle chirurgic ablation of the prechordal cells at successive stages in chick embryos generated variable holoprosencephalic phenotypes, the most severe corresponding to the earliest interventions (see above; García-Calero et al., 2008). Conditional mice were studied that are defective in *Shh* expression specifically at the whole basal hypothalamus; these still have persisting SHH signal spreading from the notochord and prechordal plate (Zhao et al., 2012; their Figure 2G). These authors showed pituitary, hypothalamic and optic defects, including reduced numbers of hormone-producing cells, a phenotype more compatible with septo-optic dysplasia than with holoprosencephalic malformations (Zhao et al., 2012). In other types of conditional mutants lacking *Shh* expression in the basal hypothalamus, this forebrain region was altered, though none of these mutants showed distinct holoprosencephalic features (Szabó et al., 2009; Shimogori et al., 2010; Corman et al., 2018). Hamdi-Rozé et al. (2020) blocked rostral (terminal) hypothalamic NOTCH effects in chick and mouse embryos, obtaining local downregulation of hypothalamic *Shh* and *Nkx2.1* expression, and alterations of other tuberal genes. These experimental embryos died shortly thereafter (after E9.5 in *Rbpj* knockout mice and after E11.5 in *Rbpj* conditional mouse mutants) so that a more detailed phenotype could not be examined.

Thus, the complexity in the spatiotemporal expression of *Shh* possibly correlates with the high variability of clinical phenotypes in holoprosencephaly or other forebrain malformations. In the normal development of the mouse, *Shh* is strongly expressed in the prechordal plate during a short neural plate period (Echelard et al., 1993). An *Shh* enhancer related specifically to prechordal *Shh* expression was reported recently (Lee et al., 2012; Sagai et al., 2019). Moreover, neural *Shh* has a dynamic spatiotemporal expression in the hypothalamic forebrain (Figure 7A). In very young mouse and chick embryos, *Shh* is first expressed in the floor plate due to the SHH signal diffused from the underlying notochord. Then *Shh* expression appears at the floor plate and expands to the hypothalamic basal plate (partly due to joint planar floor signals and frontal vertical prechordal plate effects). Basal plate *Shh* expression is secondarily downregulated at the tuberal area, the corresponding AT portion, and part of the mamillary area by repressive BMP-TBR2 signals; the tuberal area is where the dorsomedial and arcuate nuclei arise (Figure 7A; Manning et al., 2006; Alvarez-Bolado et al., 2012; Alvarez-Bolado, 2020; Trowe et al., 2013; Corman et al., 2018). Interestingly, the dorsomedial and arcuate cell populations, produced from *Shh*-downregulated areas, are gabaergic, while populations born where basal *Shh* expression persists are glutamatergic, as is the case of the ventromedial nucleus, the retromamillary area, and the mamillary area itself (Puelles et al., 2012).

*Shh* expression in the hypothalamus is required for the formation of various hypothalamic nuclei according to analysis in transgenic mice (Szabó et al., 2009; Corman et al., 2018). SHH signal secreted from extraneural and neural sources exerts its influence on target progenitor domains at the basal hypothalamus through transcription factors GLI2-GLI3, which act in a complex manner (Haddad-Tóvolli et al., 2015; Alvarez-Bolado, 2020). Very early activation of GLI1 downstream of the SHH signal activates the characteristic expression of *Nkx2.1* in the basal hypothalamus (Ruiz i Altaba, 1998). Double *Gli2/Gli3* mouse mutants showed *Shh*-downregulation in the whole basal hypothalamus (Motoyama et al., 2003). In humans, *Gli2* mutations are mostly associated with median maxillary central incisor and hypopituitarism and rarely to classic holoprosencephaly (Dubourg et al., 2016).

Furthermore, a later neural source of SHH appears separately in the prospective POA, the non-evaginated part of subpallium belonging to prosomere hp2 (Figure 7A; Flames et al., 2007; García-Calero et al., 2008; García-López et al., 2008; Bardet et al., 2010; Alvarez-Bolado et al., 2012; Puelles et al., 2016). This *Shh-expressing* secondary organizer (the subpallium organizer of Puelles, 2017) produces locally mainly preoptic astrocytes, while many *Shh-*expressing cells leave this area to populate close or distant telencephalic regions differentiating either as neurons (Marin et al., 2000; Gelman et al., 2009; Hirata et al., 2009) or as oligodendrocytes (Nery et al., 2001; Olivier et al., 2001). Note none of these two sets of authors recognized the *preoptic* locus of *Shh* expression (compare Bardet et al., 2010).

Summarizing, SHH-related alterations produced by complete or incomplete deletion of SHH sources, or defective production levels from specific extraneural or neural organizers lead to variably severe anomalies, largely shared with human SHH-related malformations with defects in the hypothalamus. More severe abnormalities (lobar holoprosencephaly) relate to insufficient SHH signaling from the prechordal plate, or inefficient response to such signals (possible case of *Six3* KO; see below). Moreover, an abnormal lack of SHH diffusion from the *Shh*-expressing preoptic subarea postulated as a *subpallial organizer* (Figure 7A; Puelles, 2017), needs to be considered to understand any subpallial anomalies present in holoprosencephaly. This alar *Shh*-expressing preoptic territory is probably induced by SHH secreted by migratory prechordal cells reaching the prospective POA at relatively late neural tube stages (García-Calero et al., 2008; Bardet et al., 2010).

### Six3

*Six3* is a transcription factor required to activate the *tuberal* basal neural *Shh* expression in the hypothalamus, besides to control directly or indirectly the expression in the forebrain of other regulative genes (e.g., *Hesx1; Foxg1, Nkx2.1, Irx3*; Lagutin et al., 2003) or activating genes that code diffusible morphogens such as FGF8 (e.g., Geng et al., 2008). In its turn, *Shh* maintains *Six3* expression in the hypothalamus (Geng et al., 2008; Jeong et al., 2008). Mutations in *Six3* are responsible for 4% of holoprosencephalic human cases (Dubourg et al., 2007; Domené et al., 2008). This is one of the first “neural” genes, i.e., genes expressed in the neural plate once the cephalic neural induction takes place, without previous expression in the mesendoderm (Oliver et al., 1995; Bovolenta et al., 1998). Hypothalamic *Six3* has rostralizing anti-*Wnt* effects, and lack of *Six3* function leads to a significant caudalization of the rostralmost forebrain (Lagutin et al., 2003). The anterior expansion of canonical *Wnt* signaling in mice embryos deficient in *Six3* causes the complete absence of the hypothalamus, eyes, and telencephalon (Lagutin et al., 2003).

At the earliest neural plate stages in the chick, the *Six3* expression ends caudally at the prospective isthmic border, thus spreading throughout the whole forebrain, though excluding the floor plate in the dorsoventral dimension (Figure 7B; Sánchez-Arrones et al., 2009; Dutra de Oliveira Melo, 2011). Thereafter, caudal *Six3* expression is progressively downregulated (apparently by antagonism with *Irx* genes), so that its caudal boundary moves progressively rostralwards through the midbrain and diencephalon. Eventually, *Six3* remains expressed only within the secondary prosencephalon, that is, the prospective hypothalamus, eye field, and telencephalon, and even appears finally restricted therein mostly to the acroterminal domain (Figure 7B; Oliver et al., 1995; Bovolenta et al., 1998; Lagutin et al., 2003; Conte et al., 2005; Geng et al., 2008; Dutra de Oliveira Melo, 2011). Loss of *Six3* function produces essentially forebrain defects rostral to the prethalamus (diencephalon), with the absence of the telencephalon, hypothalamus, eyes, and pituitary gland, as well as the olfactory placodes (Carl et al., 2002; Lagutin et al., 2003), a phenotype similar to severe holoprosencephaly phenotypes in humans (Cohen, 2006; Dubourg et al., 2007). These results also suggest that the potential effects of *Six3* upon early expressing territories caudal to the prethalamo-hypothalamic border (down to the isthmic border) are supplied by some other redundant genetic determinants. As mentioned, finally *Six3* expression is restricted to the acroterminal domain of the forebrain (i.e., rostromedial preopto-hypothalamic components; Sánchez-Arrones et al., 2009), which is postulated as a hypothalamic secondary organizer (Puelles et al., 2012; Ferran et al., 2015; Puelles, 2017). This progressive restriction of *Six3* expression to the acroterminal subdivision (though excluding its mamillary ventral portion), together with the early rostralizing influence of signaling molecules secreted by the migratory prechordal cells, might induce the significant enlargement of the terminal basal hypothalamus (tuberal area) concerning the peduncular basal hypothalamus and more caudal diencephalic basal domains. The dynamic temporospatial pattern of *Six3* expression may explain that already a *reduction* of *Six3* levels in *Six3* hypomorphic mice (≤50% signal compared to controls), is accompanied by a consequent severe downregulation of *Shh* in the hypothalamus, giving rise variously to lobar or semilobar holoprosencephalic phenotypes, depending on *Six3* dosage (Zhao et al., 2012; Geng et al., 2016). Alobar cases seem to correlate with a severe *Foxg1* downregulation mediated directly or indirectly by the decrease in the *Six3* signal (Geng et al., 2008, 2016). *Foxg1* is expressed essentially in the telencephalon (Shimogori et al., 2010), though it extends slightly into the neighboring alar paraventricular hypothalamus and the upper part of the optic stalk and retina. In conclusion, *Six3* or *Shh* null animals show a severe holoprosencephalic phenotype (with cyclopia), while diminishing variations in *Shh* and *Six3* levels result in septo-optic dysplasia or holoprosencephaly, respectively, with different degrees of severity (Jeong et al., 2008; Geng et al., 2016). These results explain the high clinical heterogeneity of both disorders.

Within the columnar model (e.g., the version of Swanson, 2012), the *Six3* lack of function data are difficult or impossible to explain since a simultaneous loss of the ventralmost “column” across the telencephalic-hypothalamic complex (Figure 6B) should have affected only the subpallium (together with the hypothalamus), and not the pallium. Moreover, failing to form the whole hypothalamus, the phenotype should have included significant pattern alterations in the remaining diencephalon. Contrarily, the latter results are normally patterned in the complete absence of its theoretic hypothalamic floor and basal components.

### *Fgf8*/FGF8

A positive feedback loop between *Fgf8* and *Shh* is also essential for normal forebrain specification (Ohkubo et al., 2002; Storm et al., 2006; Okada et al., 2008). The FGF8 protein, encoded by the *Fgf8* gene, is a member of the family of fibroblastic growth factors, whose biological activity is mediated by tyrosine kinase membrane receptors. Members of the FGFs family (FGF8; FGF15) secreted by the ANR play an important role in the development of the forebrain (Gimeno et al., 2002; Vieira et al., 2010). Less is known about the effects of FGF8 and FGF10 released by the acroterminal domain. FGF signaling molecules regulate the expression of other genes in various patterning centers (e.g., *Shh, Bmp4*, and *Wnt8b)*, and also serve non-instructively in the form of concentration gradients as positional references for regionalization of forebrain territories, thus assisting the differentiation of cell fates (Tsai et al., 2011). In the telencephalon, FGF8 secreted from the ANR organizer regulates *Six3* expression and the activation of *Nkx2.1* in the subpallium. In its turn, NKX2.1 activates *Shh* expression in the POA (the postulates subpallial organizer; Puelles, 2017), suggesting a synergistic mechanism in telencephalic development (Ohkubo et al., 2002; Geng et al., 2008). Neural *Shh* interacts with *Foxg1*, *Six3*, *Fgf8*, *Bmp4*, and *Wnt8b* for splitting the eye field and the primary unpaired telencephalic field (Geng et al., 2016). A similar interaction of these signaling pathways may be also implicated in the rostralizing patterning of the hypothalamus where most of these molecules are also expressed. In zebrafish, a decrease in both *Fgf8* and *Fgf3* produces a reduction in *Shh* expression in the basal hypothalamus (Walshe and Mason, 2003).

The* Fgf8* signal appears important in the basal acroterminal hypothalamic area (Ferran et al., 2015), particularly in the mamillary subregion. FGF8 protein spreads caudalwards from the mamillary AT source and probably affects the anteroposterior organization of the prospective mamillary/retromamillary area, and possibly also the neighboring adenohypophysis, which is likewise regionalized into distinct sectors (Figure 6). However, the impact of *Fgf8* signaling on hypothalamic development is not well understood yet either in humans or in animal models. The first recessive mutation in the *Fgf8* gene appeared in a patient with semilobar holoprosencephaly associated with diabetes insipidus and deficiencies in the pituitary hormones ACTH and TSH (McCabe et al., 2011). Recently, *Fgf8* was included among major genes implicated in holoprosencephaly due to its frequency of mutation (2.3%) in a cohort of 257 diagnosed patients, similar to the frequencies of *Six3* (2.7%) and *Gli2* (3.1%), two other holoprosencephaly-related genes (see above; Dubourg et al., 2016).

The most direct evidence for the implication of *Fgf8* signaling in the patterning of the neuroendocrine hypothalamus and the pituitary gland bases on the study of hypomorphic *Fgf8* mice with variously decreased levels of *Fgf8* expression. *Fgf8* hypomorphic homozygous mice (<50% *Fgf8* expression compared to wildtype mice) have serious defects in forebrain morphogenesis. They show prosencephalic hypoplasia with the absence of the corpus callosum, optic chiasm, and olfactory bulbs (Meyers et al., 1998; Storm et al., 2003, 2006). The severity of holoprosencephalic defects correlated with the reduction of *Fgf8* levels (Storm et al., 2006). Two main phenotypes were described: one severe phenotype shows a great reduction of adenohypophyseal tissue and absence of the posterior pituitary lobe (neurohypophysis), and a less severe phenotype, in which the pituitary gland is about normal. In the latter case, the number of oxytocinergic and vasopressinergic neurons was reduced in the hypothalamic paraventricular, supraoptic, and suprachiasmatic nuclei (Brooks et al., 2010; McCabe et al., 2011). These alterations affecting the alar hypothalamus probably result from a decrease of dorsalizing FG8 released from the ANR, rather than from the rostralizing basal acroterminal organizer (see above; Figure 6). The reduction of hypothalamic peptidergic populations appears thus linked to a decrease in the FGF8 signal. In the alar paraventricular area, the peptidergic differentiation genes are activated downstream of *Otp* and* Sim1*, which suggests a direct or indirect action of FGF8 upon the expression of these master genes. *Otp* and *Sim1* are involved in the specification *via* *Brn2* of peptidergic neurons releasing differentially oxytocin, vasopressin, corticotropin, thyrotropin, and somatostatin in the hypothalamic alar paraventricular domain (Michaud et al., 1998). Other basal peptidergic populations originate from the anterobasal area of the tuberal domain and migrate thereafter into the capsule of the ventromedial nucleus and the arcuate nucleus (Michaud et al., 1998; Acampora et al., 1999; Wang and Lufkin, 2000). FGF8 represses the *Otp1* gene in the zebrafish hypothalamus (Del Giacco et al., 2006). As mentioned above, the tuberomammillary sector of the acroterminal area also expresses strongly *Fgf8* (Figure 6A; Allen Developing Mouse Brain Atlas) and may be relevant for AP patterning of the *Otp/Sim1*-expressing perimamillary/periretromamillary basal band, where the dorsal premamillary nucleus develops (Figure 6B, green arrow).

Interestingly, in hypomorphic *Fgf8* heterozygous mice with 50% *Fgf8* expression, which have no apparent body or brain alterations, anxious behaviors were described, associated with a reduction in certain hypothalamic serotonergic populations (Brooks et al., 2014), associated with the hypothalamic ventricular organ (HVO; Figure 6A). Also, these mutants suffering stress exhibited postnatal alterations in the temporal development of corticotrophin-releasing hormone in the paraventricular hypothalamic nucleus jointly with an increase in serum cortisone levels, which suggests an alteration of the hypothalamus-pituitary-adrenal axis (Rodriguez et al., 2015).

On the other hand, Ericson et al. (1998) demonstrated in mice a requirement for FGF8 released from the hypothalamic acroterminal basal region in the maintenance of cell proliferation at the adenohypophysis *via* *Lhx3*. FGF8 is thus needed for the activation of the *Lhx3* gene and the consequent development of Rathke’s pouch (Takuma et al., 1998). The prospective adenohypophysis (a midrostral ectodermal derivative; see Sánchez-Arrones et al., 2015) is accordingly under the diffusion range of acroterminal FGF8/FGF10 signaling (Takuma et al., 1998; Ohuchi et al., 2000). However, it has not been determined yet whether the action of both factors, FGF8 and FGF10 overlap, or to what extent each of these factors modulates differentially the morphogenesis of Rathke’s pouch and the pattern of pituitary progenitors formed within it (Osmundsen et al., 2017).

### SOX2/SOX3

The transcription factors SOX2 and SOX3 are two dose-dependent regulators of hypothalamic *Shh* transcription by activation of a specific long-range *Shh* forebrain enhancer in the mouse (Zhao et al., 2012). These transcription factors are highly expressed in the hypothalamus, including the infundibulum (Wood and Episkopou, 1999; Rizzoti, 2015), and their mutations are linked to septo-optic dysplasia (Kelberman and Dattani, 2006; Sato et al., 2007). Family cases associated with mutations in *Sox2* or *Sox3* are rarely a cause of this disorder (Webb and Dattani, 2010). This disorder displays alterations in brain development with high phenotypic variability due to the contribution of multiple factors, such as genetic conditions, maternal age, and environmental factors (e.g., alcohol or other drug consumption). Patients show at least two features of the classical triad: hypoplasia of the pituitary gland, unilateral or bilateral hypoplasia in the optic nerve (occasionally with more severe abnormalities such as microphthalmia or anophthalmia), and defects in the forebrain midline (e.g., absence of the septum pellucidum or agenesis of the corpus callosum), combined or not with other defects. *Sox2* appears to be more related to anophthalmia/microphthalmia and hypogonadotropic hypogonadism, while *Sox3* tends to be associated to abnormalities of the brain midline and hypopituitarism (reviewed in Kelberman et al., 2009; Webb and Dattani, 2010).

*Sox3* knockout mice show hypopituitarism and craniofacial defects (see Kelberman et al., 2009 for a review of pituitary genetic disorders; Rizzoti and Lovell-Badge, 2007). Besides, mice with mutations in *Sox2* or *Sox3*, used as animal models of septo-optic dysplasia, display downregulation of *Shh* expression (Dattani et al., 1998; Dasen and Rosenfeld, 2001; Rizzoti et al., 2004; Kelberman and Dattani, 2006; Zhao et al., 2012). In these mutants, there is an expansion of the expression of members of the *Fgf* and *Bmp* families in the basal acroterminal domain, where the neurohypophysis is formed in contact with Rathke’s pouch. Typically, enlargement of Rathke’s pouch occurs, and multiple ectopic adenohypophysial primordia are produced (Zhao et al., 2012). Patients suffering from septo-optic dysplasia often have deficiencies in one or more pituitary hormones (Kelberman and Dattani, 2008). Furthermore, conditional elimination of *Shh* expression in the mouse basal hypothalamus showed defects consistent with septo-optic dysplasia, similarly as observed in mice embryos lacking *Sox3* and heterozygous for *Sox2*. Interestingly, *Sox2* mutants exhibited a higher reduction of *Shh* and more severe defects in eyes, hypothalamus, and pituitary gland than mutants lacking *Sox3*. These results suggest that the range of decrease of *Shh* expression levels in the hypothalamus may correlate with the phenotypic variability exhibited in patients with septo-optic dysplasia (Zhao et al., 2012). SoxB1 function controls the expression of *Six6* at the alar suprachiasmatic AT portion (Lee et al., 2012).

### Hesx1

A larger proportion of the familiar cases of septo-optic dysplasia present mutations in *Hesx1* in homozygosis or heterozygosis (latter type related to milder phenotypes; Dattani et al., 1998; Thomas et al., 2001; Kelberman and Dattani, 2007a,b). There is, however, a low incidence in humans (1% of cases with septo-optic dysplasia, or presence of a single feature of the syndromic triad; McNay et al., 2007). *Hesx1* null mice exhibit midline forebrain and eye defects, as well as pituitary dysplasia, highly comparable to human septo-optic dysplasia (Dattani et al., 1998; Andoniadou et al., 2007; Sajedi et al., 2008). *Hesx1* is a paired-like homeobox gene with a spatiotemporal dynamic expression, which acts as a transcriptional repressor. However, its targets remain unknown. In the mouse, this gene is expressed first during gastrulation in the anterior visceral endoderm, and later in the prechordal mesoderm and rostromedial forebrain (at neural plate stages). The neural expression domain, descending dorsoventrally into the hypothalamus along with the median acroterminal domain, roughly overlaps that of *Six3*. Ulteriorly, *Hesx1* expression becomes restricted to Rathke’s pouch, and its signal disappears at E13.5 (Hermesz et al., 1996; Thomas and Beddington, 1996). Other genes related to septo-optic dysplasia are *Tcf4*, *Noggin*, *Wnt5a*, and *Vax1* (Bertuzzi et al., 1999; Brinkmeier et al., 2007; Davis and Camper, 2007; Potok et al., 2008). SHH released from the prechordal plate regulates the expression of *Vax1* and *Vax2* in the alar hypothalamus. These are genes implicated in the development of the eye, alar hypothalamus, and prethalamus (Take-uchi et al., 2003; Kim and Lemke, 2006).

## Concluding Remarks

In summary, experimental evidence in different animal models, complemented with mutations found in humans related to forebrain and craniofacial defects, show the implication of several signaling molecules (mainly SHH, FGF8, BMPs, possibly also WNT8b) and transcription factors (e.g., GLI1-3, HESX1, SIX3, SOX2, SOX3), in the specification of complex rostral forebrain structure [hypothalamus, eyes, telencephalon; see a recent analysis by Kim et al. (2019) of familiar combinatorial genetic and phenotypic precedents]. These molecules execute their respective roles acting upstream or downstream of *spatially distinct* signaling pathways able to organize DV or AP patterns, and acting in a parallel or sequential manner. Disruptions of one or more signaling molecules secreted by such organizer sources can generate abnormal development of the forebrain (including the hypothalamus). Mutations or biochemical disturbances caused by environmental factors may alter the functional expression or dosage of both transcription factor genes and/or morphogens, hence underpinning different forebrain disorders showing considerable clinical heterogeneity.

The strict dependency of causal explanations on the accurate morphological assessment of DV vs. AP positions has determined the recent paradigm shift from the classic columnar morphological model to the prosomeric model. The columnar model served conventionally as long as no causal explanation was possible and the field mainly demanded superficial functional analysis. As soon as the molecular era arrived and experimental developmental neurobiology started, it became clear that the outdated columnar model produces misleading and confusing causal interpretations, mainly due to its arbitrary definition of the axial dimension of the forebrain. The shift to the causally more realistic prosomeric model poses no problem for the integration of the advances in functional neurobiology made during the preceding columnar era.

## Author Contributions

CD: conceptualization, writing, and figures. LP: conceptualization, writing, and revision. All authors contributed to the article and approved the submitted version.

## Conflict of Interest

The authors declare that the research was conducted in the absence of any commercial or financial relationships that could be construed as a potential conflict of interest.
